# Supplementary material for: What Do We Know about Social and Non-Social Factors Influencing the Pathway from Cognitive Health to Dementia? A Systematic Review of Reviews
Source: Brain Sci. 2022 Sep 8;12(9):1214. doi: 10.3390/brainsci12091214 (PMC9497077; doi:10.3390/brainsci12091214)
Supplement: Supplementary file 1 [file brainsci-12-01214-s001.zip › List S1. Full references.pdf]

Lenart-Bugla et al., 2022: What do we know about social and non-social factors influencing the pathway from cognitive health to dementia? A systematic review of reviews.

Supplementary material

List 1. References of included Systematic Reviews and Meta-analyses.

Category "Somatic Diseases & Biological"

Agarwal et al., 2014; Ali et al., 2018; Allan and Ebmeier, 2011; Almeida et al., 2018; Angelousi et al., 2014; Annweiler et al., 2016; Assuncao et al., 2018; Atti et al., 2019; Autier et al., 2014; Azarpazhooh et al., 2018; Beauchet et al., 2018, 2014; Beydoun et al., 2014; Blom et al., 2013; Bolandzadeh et al., 2012; Borges et al., 2019; Brundel et al., 2012; Bubu et al., 2017; Canevelli et al., 2014; Cao et al., 2016; Catindig et al., 2012; W. Chang et al., 2013; X.-L. Chang et al., 2013; Charidimou et al., 2018; H. Chen et al., 2018; J. Chen et al., 2018; Chen et al., 2016; Cheng et al., 2014, 2012; Cipolli et al., 2019; Cooper et al., 2015; Crichton et al., 2012; Crocker et al., 2016; Dahl and Hassing, 2013; Danna et al., 2016; Davies et al., 2015; de Almondes et al., 2016; Deckers et al., 2017, 2014; Dobbels et al., 2019; Dong et al., 2017, 2018; Du et al., 2016; Elias-Sonnenschein et al., 2011; Etgen et al., 2012a; Fang et al., 2018; Fei and Jianhua, 2013; Feinkohl et al., 2018; Fink et al., 2015; A. H. Ford et al., 2018; E. Ford et al., 2018; Forero et al., 2016; Galbiati et al., 2019; Gao et al., 2016; Goodwill and Cassandra, 2017; Gore et al., 2014; Guan et al., 2012, 2011; Guerchet et al., 2011; Haaksma et al., 2017; Hagg et al., 2017; Hajduk et al., 2013; Hao et al., 2011; He et al., 2013; Henneghan, 2016; Heringa et al., 2013; Hersi et al., 2017; Hua et al., 2012, 2011; Huang et al., 2017; Hui et al., 2015; Jiang et al., 2019; Jin et al., 2012; Julien et al., 2017; Jun et al., 2010; Keage et al., 2009; Klimova et al., 2018; Kobayashi-Cuya et al., 2018; Kopf and Frölich, 2009; Koyama et al., 2013, 2012; Kueper et al., 2017; Kunkle et al., 2019; Kuzma et al., 2018; Kwok et al., 2011; Lai et al., 2016; Lam et al., 2017; Lancaster et al., 2017; Lee et al., 2010; Lekoubou et al., 2014; Leng et al., 2017; Lennon et al., 2019; J. Li et al., 2017; Li et al., 2015, 2013; Y.-Q. Li et al., 2016; Y. Li et al., 2017; Lin et al., 2014, 2012; B. Liu et al., 2012; Liu et al., 2010, 2009, 2013; X. Liu et al., 2012; Y. Liu et al., 2016; Loef et al., 2012; M Loef and Walach, 2013; Martin Loef and Walach, 2012; Lu et al., 2009, 2016, 2014; Lucchetta et al., 2018; Luck et al., 2010; Lv et al., 2016; Ma et al., 2014; Makin et al., 2013; Manley et al., 2017; Mattishent and Loke, 2016; McGuinness et al., 2009; Meade et al., 2018; Moran et al., 2014; Moroni et al., 2016; Naismith and Mowszowski, 2018; Ojagbemi and Ffytche, 2016; Oldham et al., 2018; Pal et al., 2018; Pan and Kastin, 2014; Pase et al., 2012; Pendlebury and Rothwell, 2009; Plassman et al., 2010; Power et al., 2011; Proietti et al., 2014; Purnell et al., 2009; Qin et al., 2012; Quinn et al., 2011; Rai, 2017; Rayes et al., 2018; Rensma et al., 2018; Ryman et al., 2014; Sakusic et al., 2018; Savva et al., 2010; Seifan et al., 2015; Seitz et al., 2011; Sekhon et al., 2019; Sharp et al., 2011; Shen et al., 2017; Shi et al., 2018, 2015; Siervo et al., 2011; Solfrizzi et al., 2017; Sommer et al., 2017; Song et al., 2018; Spano et al., 2015; Stacey et al., 2017; Stefanidis et al., 2018; Stewart et al., 2015; Stocker et al., 2018; Su et al., 2017; Subota et al., 2017; Sun et al., 2015, 2019; Tonsekar et al., 2017; Udompanich et al., 2013; Ungprasert et al., 2016; Vagelatos et al., 2013; Van Dam and Van Gool, 2009; van den Berg et al., 2009; van der Velpen et al., 2017; van Rijsbergen et al., 2014; van Rooij et al., 2016; van Sloten et al., 2015; Veronese et al., 2016; C. Wang et al., 2014; J. Wang et al., 2018; Q. Wang et al., 2019; X. Wang et al., 2014; Y.-C. Wang et al., 2018; Wang et al., 2017, 2013, 2016; Weber et al., 2019; Wu et al., 2013; Wu and Nakanishi, 2014; H.-M. Xu et al., 2017; Yang et al., 2014; Yuan et al., 2018, 2015; Yuan and Wang, 2017; D.-M. Zhang et al., 2017; J. Zhang et al., 2017; X. Zhang et al., 2016; Zheng et al., 2018, 2017; Zhong et al., 2015; Zhou and Chen, 2019; Zhou et al., 2015, 2014, 2019; Zhu et al., 2017

Category "Lifestyle"

Aarsland et al., 2010; Anderson et al., 2014; Anstey et al., 2009, 2011; Arab et al., 2013; Aridi et al., 2017; Attuquayefio and Stevenson, 2015; Beydoun et al., 2014; Blom et al., 2013; Canevelli et al., 2016; Cao et al., 2016; Catindig et al., 2012; Chapko et al., 2017; Chen et al., 2013b, 2013a; Cheng et al., 2017; Cooper et al., 2015; Crichton et al., 2013; Dangour et al., 2010; de Assis and de Almondes, 2017; Deckers et al., 2014; Di Marco et al., 2014; Dyer et al., 2019; Forbes et al., 2015; E. Ford et al., 2018; Fotuhi et al., 2009; Guure et al., 2017; Hamer and Chida, 2009; Hersi et al., 2017; Hill et al., 2019; Huckans et al., 2013; Iizuka et al., 2019; Jia et al., 2019; Kivimäki et al., 2019; Krishna et al., 2019; Kuzma et al., 2018; Lafortune et al., 2016; Last et al., 2017; Lee, 2018; Lee et al., 2010; Lehert et al., 2015; Lekoubou et al., 2014; Liapis and Harding, 2017; Q.-P. Liu et al., 2016; Lo et al., 2016; Loef et al., 2012; M Loef and Walach, 2012; Martin Loef and Walach, 2012, 2013; Ma et al., 2016; Meng et al., 2014; Munoz Fernandez et al., 2017; Nader and Sanchez, 2018; Niu et al., 2018; Pedditizi et al., 2016; Perez et al., 2012; Petersson and Philippou, 2016; Piazza-Gardner et al., 2013; Plassman et al., 2010; Purnell et al., 2009; Sajeev et al., 2016; Siervo et al., 2011; Snowden et al., 2011; Solfrizzi et al., 2017; Stephen et al., 2017; Stewart et al., 2015; Stirland et al., 2018; Tada and Miura, 2017; van de Rest et al., 2015; Vnukova et al., 2017; Wang et al., 2012; L Wu et al., 2017; Lei Wu et al., 2017; Wu et al., 2018; S. Wu et al., 2015; W. Xu et al., 2017; Yates et al., 2016; Yusufov et al., 2017; Zeng et al., 2017; D.-M. Zhang et al., 2017; Y. Zhang et al., 2016; Zhong et al., 2015

Category "Social"

Besser et al., 2017; Boss et al., 2015; Desai et al., 2020; Di Marco et al., 2014; Hersi et al., 2017; Iizuka et al., 2019; Kelly et al., 2017; Kuiper et al., 2016, 2015; Lafortune et al., 2016; Lekoubou et al., 2014; McGrattan et al., 2021; Penninkilampi et al., 2018; Plassman et al., 2010; Sommerlad et al., 2017; Walker et al., 2020; Wu, Z. et al., 2020

Category "Psychiatric & Psychological"

Bastida et al., 2019; Becker et al., 2018; Chan et al., 2019; Chapko et al., 2017; Cherbuin et al., 2015; Childress et al., 2013; Cooper et al., 2015; da Silva et al., 2013; Danna et al., 2016; Deckers et al., 2014; Deligkaris et al., 2014; Diniz et al., 2017, 2013; E. Ford et al., 2018; Forlenza et al., 2013; Gao et al., 2013; Gimson et al., 2018; Gore et al., 2014; Gulpers et al., 2016; Hasselbalch et al., 2011; Hersi et al., 2017; Killin et al., 2016; Lekoubou et al., 2014; J.-Q. Li et al., 2016; Low et al., 2013; MacLullich et al., 2009; Mendonca et al., 2016; Mourao et al., 2016; Plassman et al., 2010; Santabárbara et al., 2019; Song et al., 2018; Tan et al., 2019; van Dalen et al., 2018; van Rijsbergen et al., 2014; X. J. Wang et al., 2019; Yates et al., 2013

Lenart-Bugla et al., 2022: What do we know about social and non-social factors influencing the pathway from cognitive health to dementia? A systematic review of reviews.

Category "Socioeconomic"

Besser et al., 2017; Beydoun et al., 2014; Chapko et al., 2017; Cheng et al., 2016; Ennis et al., 2015; E. Ford et al., 2018; Hersi et al., 2017; Kuzma et al., 2018; Lekoubou et al., 2014; J.-Q. Li et al., 2016; Luck et al., 2010; McCulloch et al., 2016; Meng and D'Arcy, 2012; Oliveira et al., 2019; Plassman et al., 2010; Sharp and Gatz, 2011; Song et al., 2018; X.-J. Wang et al., 2019; Y.-T. Wu et al., 2015

Category "Environmental"

Cao et al., 2016; Clifford et al., 2016; de Keijzer et al., 2016; Dimakakou et al., 2018; Du et al., 2017; Etgen et al., 2012b; Gunnarsson and Bodin, 2019; Hersi et al., 2017; Jalilian et al., 2018; Killin et al., 2016; Peters et al., 2015; Plassman et al., 2010; Shen and Ji, 2015; Sommer et al., 2017; X.-J. Wang et al., 2019; Yan et al., 2016

Category "Demographic"

Besser et al., 2017; Catindig et al., 2012; X.-L. Chang et al., 2013; Chapko et al., 2017; Crean et al., 2011; Hersi et al., 2017; Hu et al., 2017; Lekoubou et al., 2014; Luck et al., 2010; Morra et al., 2013; Mukadam et al., 2017; Ryman et al., 2014; Song et al., 2018; Y.-T. Wu et al., 2015; X. and Y., 2018; J. Zhang et al., 2017

Full References

Aarsland, D., Sardahaee, F.S., Anderssen, S., Ballard, C., group, A.S.S.R., 2010. Is physical activity a potential preventive factor for vascular dementia? A systematic review. Aging Ment. Health 14, 386–395. https://doi.org/https://dx.doi.org/10.1080/13607860903586136

Agarwal, R., Tripathi, C.B., CB, T., Tripathi, C.B., CB, T., Tripathi, C.B., 2014. Association of Apolipoprotein E Genetic Variation in Alzheimer's Disease in Indian Population: A Meta-Analysis. Am. J. Alzheimers. Dis. Other Demen. 29, 575–582. https://doi.org/10.1177/1533317514531443

Ali, J.I., Smart, C.M., Gawryluk, J.R., 2018. Subjective cognitive decline and APOE epsilon4: A systematic review. J. Alzheimer's Dis. 65, 303–320. https://doi.org/http://dx.doi.org/10.3233/JAD-180248

Allan, C.L., Ebmeier, K.P., 2011. The influence of ApoE4 on clinical progression of dementia: a meta-analysis. Int. J. Geriatr. Psychiatry 26, 520–526. https://doi.org/https://dx.doi.org/10.1002/gps.2559

Almeida, J.F.F., Dos Santos, L.R., Trancozo, M., de Paula, F., 2018. Updated Meta-Analysis of BIN1, CR1, MS4A6A, CLU, and ABCA7 Variants in Alzheimer's Disease. J. Mol. Neurosci. 64, 471–477. https://doi.org/https://dx.doi.org/10.1007/s12031-018-1045-y

Anderson, D., Seib, C., Rasmussen, L., 2014. Can physical activity prevent physical and cognitive decline in postmenopausal women? A systematic review of the literature. Maturitas 79, 14–33. https://doi.org/10.1016/j.maturitas.2014.06.010

Angelousi, A., Girerd, N., Benetos, A., Frimat, L., Gautier, S., Weryha, G., Boivin, J.-M., 2014. Association between orthostatic hypotension and cardiovascular risk, cerebrovascular risk, cognitive decline and falls as well as overall mortality: a systematic review and meta-analysis. J. Hypertens. 32, 1562–1571. https://doi.org/https://dx.doi.org/10.1097/HJH.0000000000000235

Annweiler, C., Milea, D., Whitson, H.E., Cheng, C.-Y., Wong, T.-Y., Ikram, M.K., Lamoureux, E.L., Sabanayagam, C., 2016. Vitamin D insufficiency and cognitive impairment in Asians: a multi-ethnic population-based study and meta-analysis. J. Intern. Med. 280, 300–311. https://doi.org/https://dx.doi.org/10.1111/joim.12491

Anstey, K.J., Cherbuin, N., Budge, M., Young, J., 2011. Body mass index in midlife and late-life as a risk factor for dementia: a meta-analysis of prospective studies. Obes. Rev. 12, e426-37. https://doi.org/10.1111/j.1467-789X.2010.00825.x

Anstey, K.J., Mack, H.A., Cherbuin, N., 2009. Alcohol Consumption as a Risk Factor for Dementia and Cognitive Decline: Meta-Analysis of Prospective Studies. Am. J. Geriatr. Psychiatry 17, 542–555. https://doi.org/10.1097/JGP.0b013e3181a2fd07

Arab, L., Khan, F., Lam, H., 2013. Epidemiologic evidence of a relationship between tea, coffee, or caffeine consumption and cognitive decline. Adv. Nutr. 4, 115–122. https://doi.org/https://dx.doi.org/10.3945/an.112.002717

Aridi, Y.S., Walker, J.L., Wright, O.R.L., 2017. The Association between the Mediterranean Dietary Pattern and Cognitive Health: A Systematic Review. Nutrients 9. https://doi.org/https://dx.doi.org/10.3390/nu9070674

Assuncao, N., Sudo, F.K., Drummond, C., de Felice, F.G., Mattos, P., 2018. Metabolic Syndrome and cognitive decline in the elderly: A systematic review. PLoS One 13, e0194990. https://doi.org/https://dx.doi.org/10.1371/journal.pone.0194990

Atti, A.R., Valente, S., Iodice, A., Caramella, I., Ferrari, B., Albert, U., Mandelli, L., De Ronchi, D., 2019. Metabolic syndrome, mild cognitive impairment, and dementia: A meta-analysis of longitudinal studies. Am. J. Geriatr. Psychiatry 27, 625–637. https://doi.org/http://dx.doi.org/10.1016/j.jagp.2019.01.214

Attuquayefio, T., Stevenson, R.J., 2015. A systematic review of longer-term dietary interventions on human cognitive function: Emerging patterns and future directions. Appetite 95, 554–570. https://doi.org/https://dx.doi.org/10.1016/j.appet.2015.08.023

Autier, P., Boniol, M., Pizot, C., Mullie, P., 2014. Vitamin D status and ill health: a systematic review. lancet. Diabetes Endocrinol., [Comment in: Lancet Diabetes Endocrinol. 2014 Jan;2(1):1; PMID: 24622652 [https://www.ncbi.nlm.nih.gov/pubmed/24622652]][Comment in: Lancet Diabetes Endocrinol. 2014 Apr;2(4):273-4; PMID: 24703040 [https://www.ncbi.nlm.nih.gov/pubmed/24703040]][Comment in 2, 76–89. https://doi.org/https://dx.doi.org/10.1016/S2213-8587(13)70165-7

Azarpazhooh, M.R., Avan, A., Cipriano, L.E., Munoz, D.G., Sposato, L.A., Hachinski Lauren E.; ORCID: http://orcid.org/0000-0001-5568-4516, V.A.I.-O. http://orcid. org/Ciprian., 2018. Concomitant vascular and neurodegenerative pathologies double the risk of dementia. Alzheimer's Dement. J. Alzheimer's Assoc. 14, 148–156. https://doi.org/http://dx.doi.org/10.1016/j.jalz.2017.07.755

Bastida, J.D., Pujol, J.G., Vives, S.V., Font, S.J., Eickhoff, A.F., Cardona, E.A., 2019. Disorder of the personality: a possible factor of risk for the dementia. Actas Esp. Psiquiatr. 47, 61–69.

Beauchet, O., Allali, G., Montero-Odasso, M., Sejdic, E., Fantino, B., Annweiler, C., 2014. Motor phenotype of decline in cognitive performance among community-dwellers without dementia: population-based study and meta-analysis. PLoS One 9, e99318. https://doi.org/https://dx.doi.org/10.1371/journal.pone.0099318

Beauchet, O., Sekhon, H., Barden, J., Liu-Ambrose, T., Chester, V.L., Szturm, T., Grenier, S., Leonard, G., Bherer, L., Allali, G., 2018. Association of motoric cognitive risk syndrome with cardiovascular disease and risk factors: Results from an original study and meta-analysis. J. Alzheimer's Dis. 64, 875–887. https://doi.org/http://dx.doi.org/10.3233/JAD-180203

Becker, E., CL, O.R., Lahmann, C., Rücker, G., Bauer, J., Boeker, M., 2018. Anxiety as a risk factor of Alzheimer's disease and vascular dementia. Br. J. Psychiatry 213, 654–660. https://doi.org/10.1192/bjp.2018.173

Lenart-Bugla et al., 2022: What do we know about social and non-social factors influencing the pathway from cognitive health to dementia? A systematic review of reviews.

Besser, L.M., McDonald, N.C., Song, Y., Kukull, W.A., Rodriguez, D.A., 2017. Neighborhood environment and cognition in older adults: A systematic review. Am. J. Prev. Med. 53, 241–251. https://doi.org/http://dx.doi.org/10.1016/j.amepre.2017.02.013

Beydoun, M.A., Beydoun, H.A., Gamaldo, A.A., Teel, A., Zonderman, A.B., Wang, Y., 2014. Epidemiologic studies of modifiable factors associated with cognition and dementia: systematic review and meta-analysis. BMC Public Health 14, 643. https://doi.org/https://dx.doi.org/10.1186/1471-2458-14-643

Blom, K., Emmelot-Vonk, M.H., Koek, H. (Dineke) L., 2013. The influence of vascular risk factors on cognitive decline in patients with dementia: A systematic review. Maturitas 76, 113–117. https://doi.org/http://dx.doi.org/10.1016/j.maturitas.2013.06.011

Bolandzadeh, N., Davis, J.C., Tam, R., Handy, T.C., Liu-Ambrose, T., 2012. The association between cognitive function and white matter lesion location in older adults: A systematic review. BMC Neurol. 12.

Borges, M.K., Canevelli, M., Cesari, M., Aprahamian, I., 2019. Frailty as a Predictor of Cognitive Disorders: A Systematic Review and Meta-Analysis. Front. Med. 6, 26. https://doi.org/https://dx.doi.org/10.3389/fmed.2019.00026

Boss, L., Kang, D.-H., Branson, S., 2015. Loneliness and cognitive function in the older adult: A systematic review. Int. Psychogeriatrics 27, 541–553. https://doi.org/http://dx.doi.org/10.1017/S1041610214002749

Brundel, M., de Bresser, J., van Dillen, J.J., Kappelle, L.J., Biessels, G.J., 2012. Cerebral microinfarcts: a systematic review of neuropathological studies. J. Cereb. Blood Flow Metab. 32, 425–436. https://doi.org/https://dx.doi.org/10.1038/jcbfm.2011.200

Bubu, O.M., Brannick, M., Mortimer, J., Umasabor-Bubu, O., Sebastiao, Y. V, Wen, Y., Schwartz, S., Borenstein, A.R., Wu, Y., Morgan, D., Anderson, W.M., 2017. Sleep, Cognitive impairment, and Alzheimer's disease: A Systematic Review and Meta-Analysis. Sleep 40. https://doi.org/https://dx.doi.org/10.1093/sleep/zsw032

Canevelli, M., Lucchini, F., Quarata, F., Bruno, G., Cesari, M., 2016. Nutrition and Dementia: Evidence for Preventive Approaches?. Nutrients 8, 144. https://doi.org/https://dx.doi.org/10.3390/nu8030144

Canevelli, M., Piscopo, P., Talarico, G., Vanacore, N., Blasimme, A., Crestini, A., Tosto, G., Troili, F., Lenzi, G.L., Confaloni, A., Bruno Giuseppe; ORCID: http://orcid.org/0000-0001-6948-7169, G.A.I.-O. http://orcid. org/Brun., 2014. Familial Alzheimer's disease sustained by presenilin 2 mutations: Systematic review of literature and genotype-phenotype correlation. Neurosci. Biobehav. Rev. 42, 170–179. https://doi.org/http://dx.doi.org/10.1016/j.neubiorev.2014.02.010

Cao, L., Tan, L., Wang, H.-F., Jiang, T., Zhu, X.-C., Lu, H., Tan, M.-S., Yu, J.-T., 2016. Dietary patterns and risk of dementia: A systematic review and meta-analysis of cohort studies. Mol. Neurobiol. 53, 6144–6154. https://doi.org/http://dx.doi.org/10.1007/s12035-015-9516-4

Catindig, J.-A.S., Venketasubramanian, N., Ikram, M.K., Chen, C., 2012. Epidemiology of dementia in Asia: Insights on prevalence, trends and novel risk factors. J. Neurol. Sci. 321, 11–16. https://doi.org/http://dx.doi.org/10.1016/j.jns.2012.07.023

Chan, J., Yiu, K., Kwok, T., Wong, S., Tsoi, K., 2019. Depression and Antidepressants as Potential Risk Factors in Dementia: A Systematic Review and Meta-analysis of 18 Longitudinal Studies. J. Am. Med. Dir. Assoc. 20, 279. https://doi.org/10.1016/j.jamda.2018.12.004

Chang, W., Zhang, L., Jin, Y., Yao, Y., 2013. Meta-analysis of the transforming growth factor-β1 polymorphisms and susceptibility to Alzheimer's disease. J. Neural Transm. 120, 353–360. https://doi.org/10.1007/s00702-012-0850-7

Chang, X.-L., Zhou, H.-Q., Lei, C.-Y., Wu, B., Chen, Y.-C., Hao, Z.-L., Dong, W., Liu, M., XL, C., HQ, Z., CY, L., Wu, B., YC, C., ZL, H., Dong, W., Liu, M., Chang, X.-L., Zhou, H.-Q., Lei, C.-Y., Wu, B., Chen, Y.-C., Hao, Z.-L., Dong, W., Liu, M., XL, C., HQ, Z., CY, L., Wu, B., YC, C., ZL, H., Dong, W., Liu, M., 2013. Association between asymptomatic carotid stenosis and cognitive function: A systematic review. Neurosci. Biobehav. Rev. 37, 1493–1499. https://doi.org/10.1016/j.neubiorev.2013.05.011

Chapko, D., McCormack, R., Black, C., Staff, R., Murray, A., 2017. Life-course determinants of cognitive reserve (CR) in cognitive aging and dementia - a systematic literature review. Aging Ment. Health 22, 915–926. https://doi.org/10.1080/13607863.2017.1348471

Charidimou, A., Shams, S., Romero, J.R., Ding, J., Veltkamp, R., Horstmann, S., Eiriksdottir, G., van Buchem, M.A., Gudnason, V., Himali, J.J., Gurol, M.E., Viswanathan, A., Imaizumi, T., Vernooij, M.W., Seshadri, S., Greenberg, S.M., Benavente, O.R., Launer, L.J., Shoamanesh, A., Initiative, I.M.-M., 2018. Clinical significance of cerebral microbleeds on MRI: A comprehensive meta-analysis of risk of intracerebral hemorrhage, ischemic stroke, mortality, and dementia in cohort studies (v1). Int. J. Stroke 13, 454–468. https://doi.org/https://dx.doi.org/10.1177/1747493017751931

Chen, H., Xue, W., Li, J., Fu, K., Shi, H., Zhang, B., Teng, W., Tian, L., 2018. 25-hydroxyvitamin D levels and the risk of dementia and Alzheimer's disease: A dose-response meta-analysis. Front. Aging Neurosci. 10. https://doi.org/http://dx.doi.org/10.3389/fnagi.2018.00368

Chen, J., Ren, C.-J., Wu, L., Xia, L.-Y., Shao, J., Leng, W.-D., Zeng, X.-T., 2018. Tooth loss is associated with increased risk of dementia and with a dose-response relationship. Front. Aging Neurosci. 10. https://doi.org/http://dx.doi.org/10.3389/fnagi.2018.00415

Chen, Q., Liang, B., Wang, Z., Cheng, X., Huang, Y., Liu, Y., Huang, Z., 2016. Influence of four polymorphisms in ABCA1 and PTGS2 genes on risk of Alzheimer's disease: a meta-analysis. Neurol. Sci. 37, 1209–1220. https://doi.org/10.1007/s10072-016-2579-9

Chen, R., Clifford, A., Lang, L., Anstey, K.J., 2013a. Is exposure to secondhand smoke associated with cognitive parameters of children and adolescents? A systematic literature review. Ann. Epidemiol. 23, 652–661. https://doi.org/https://dx.doi.org/10.1016/j.annepidem.2013.07.001

Chen, R., Hu, Z., Orton, S., Chen, R.-L., Wei Sophie; ORCID: http://orcid.org/0000-0002-8577-216X, L.A.I.-O. http://orcid. org/Orto., 2013b. Association of passive smoking with cognitive impairment in nonsmoking older adults: A systematic literature review and a new study of Chinese cohort. J. Geriatr. Psychiatry Neurol. 26, 199–208. https://doi.org/http://dx.doi.org/10.1177/0891988713496165

Cheng, C., Huang, C.-L., Tsai, C.-J., Chou, P.-H., Lin, C.-C., Chang, C.-K., C., C., C.-L., H., C.-J., T., P.-H., C., C.-C., L., C.-K., C., Cheng, C., Huang, C.-L., Tsai, C.-J., Chou, P.-H., Lin, C.-C., Chang, C.-K., 2017. Alcohol-Related Dementia: A Systemic Review of Epidemiological Studies. Psychosomatics 58, 331–342. https://doi.org/http://dx.doi.org/10.1016/j.psym.2017.02.012

Cheng, D., Liang, B., Hao, Y., Zhou, W., 2014. Estrogen receptor alpha gene polymorphisms and risk of Alzheimer's disease: evidence from a meta-analysis. Clin. Interv. Aging 9, 1031–1038. https://doi.org/https://dx.doi.org/10.2147/CIA.S65921

Cheng, G., Huang, C., Deng, H., Wang, H., 2012. Diabetes as a risk factor for dementia and mild cognitive impairment: a meta-analysis of longitudinal studies. Intern. Med. J. 42, 484–491. https://doi.org/10.1111/j.1445-5994.2012.02758.x

Cheng, H.G., Shidhaye, R., Charlson, F., Deng, F., Lyngdoh, T., Chen, S., Nanda, S., Lacroix, K., Baxter, A., Whiteford, H., 2016. Social correlates of mental, neurological, and substance use disorders in China and India: a review. The lancet. Psychiatry 3, 882–899. https://doi.org/https://dx.doi.org/10.1016/S2215-0366(16)30166-3

Cherbuin, N., Kim, S., Anstey, K.J., 2015. Dementia risk estimates associated with measures of depression: a systematic review and meta-analysis. BMJ Open 5, e008853. https://doi.org/https://dx.doi.org/10.1136/bmjopen-2015-008853

Childress, J.E., McDowell, E.J., Dalai, V.V.K., Bogale, S.R., Ramamurthy, C., Jawaid, A., Kunik, M.E., Qureshi, S.U., Schulz, P.E., 2013. Hippocampal volumes in patients with chronic combat-related posttraumatic stress disorder: A systematic review. J. Neuropsychiatry Clin. Neurosci. 25, 12–25. https://doi.org/http://dx.doi.org/10.1176/appi.neuropsych.12010003

Cipolli, C.G., Yassuda, S.M., Aprahamian, I., 2019. Sarcopenia Is Associated with Cognitive Impairment in Older Adults: A Systematic Review and Meta-Analysis. J. Nutr. Health Aging 23, 525–531. https://doi.org/10.1007/s12603-019-1188-8

Clifford, A., Lang, L., Chen, R., Anstey, K.J., Seaton, A., 2016. Exposure to air pollution and cognitive functioning across the life course--A systematic literature review. Environ. Res. 147, 383–398. https://doi.org/https://dx.doi.org/10.1016/j.envres.2016.01.018

Cooper, C., Sommerlad, A., Lyketsos, C.G., Livingston Claudia; ORCID: http://orcid.org/0000-0002-2777-7616, G.A.I.-O. http://orcid. org/Coope., 2015. Modifiable predictors of dementia in mild cognitive impairment: A systematic review and meta-analysis. Am. J. Psychiatry, American Journal of Insanity 172, 323–334. https://doi.org/http://dx.doi.org/10.1176/appi.ajp.2014.14070878

Crean, S., Ward, A., CJ, M., JM, C., MN, C., NL, B., HM, A., 2011. Apolipoprotein e ε4 prevalence in alzheimer's disease patients varies across global populations: a systematic literature review and meta-analysis. Dement. Geriatr. Cogn. Disord. 31, 20–30. https://doi.org/10.1159/000321984

Crichton, G.E., Bryan, J., Murphy, K.J., 2013. Dietary antioxidants, cognitive function and dementia--a systematic review. Plant Foods Hum. Nutr. 68, 279–292. https://doi.org/https://dx.doi.org/10.1007/s11130-013-0370-0

Crichton, G.E., Elias, M.F., Buckley, J.D., Murphy, K.J., Bryan, J., Frisardi, V., 2012. Metabolic syndrome, cognitive performance, and dementia. J. Alzheimers. Dis. 30 Suppl 2, S77-87. https://doi.org/https://dx.doi.org/10.3233/JAD-2011-111022

Crocker, E., Beggs, T., Hassan, A., Denault, A., Lamarche, Y., Bagshaw, S., Elmi-Sarabi, M., Hiebert, B., Macdonald, K., Giles-Smith, L., Tangri, N., Arora, R.C., 2016. Long-Term Effects of Postoperative Delirium in Patients Undergoing Cardiac Operation: A Systematic Review. Ann. Thorac. Surg. 102, 1391–1399. https://doi.org/https://dx.doi.org/10.1016/j.athoracsur.2016.04.071

da Silva, J., Goncalves-Pereira, M., Xavier, M., Manuel, M.-L., 2013. Affective disorders and risk of developing dementia: Systematic review. Br. J. Psychiatry, Journal of Mental Science, The Asylum Journal, The Asylum Journal of Mental Science 202, 177–186. https://doi.org/http://dx.doi.org/10.1192/bjp.bp.111.101931

Dahl, A.K., Hassing, L.B., 2013. Obesity and cognitive aging. Epidemiol. Rev. 35, 22–32. https://doi.org/https://dx.doi.org/10.1093/epirev/mxs002

Dangour, A.D., Whitehouse, P.J., Rafferty, K., Mitchell, S.A., Smith, L., Hawkesworth, S., Vellas, B., 2010. B-vitamins and fatty acids in the prevention and treatment of Alzheimer's disease and dementia: A systematic review. J. Alzheimer's Dis. 22, 205–224.

Danna, S.M., Graham, E., Burns, R.J., Deschenes, S.S., Schmitz, N., 2016. Association between Depressive Symptoms and Cognitive Function in Persons with Diabetes Mellitus: A Systematic Review. PLoS One 11, e0160809. https://doi.org/https://dx.doi.org/10.1371/journal.pone.0160809

Davies, G., Armstrong, N., Bis, J.C., Bressler, J., Chouraki, V., Giddaluru, S., Hofer, E., Ibrahim-Verbaas, C.A., Kirin, M., Lahti, J., van der Lee, S.J., Le Hellard, S., Liu, T., Marioni, R.E., Oldmeadow, C., Postmus, I., Smith, A. V, Smith, J.A., Thalamuthu, A., Thomson, R., Vitart, V., Wang, J., Yu, L., Zgaga, L., Zhao, W., Boxall, R., Harris, S.E., Hill, W.D., Liewald, D.C., Luciano, M., Adams, H., Ames, D., Amin, N., Amouyel, P., Assareh, A.A., Au, R., Becker, J.T., Beiser, A., Berr, C., Bertram, L., Boerwinkle, E., Buckley, B.M., Campbell, H., Corley, J., De Jager, P.L., Dufouil, C., Eriksson, J.G., Espeseth, T., Faul, J.D., Ford, I., Scotland, G., Gottesman, R.F., Griswold, M.E., Gudnason, V., Harris, T.B., Heiss, G., Hofman, A., Holliday, E.G., Huffman, J., Kardia, S.L.R., Kochan, N., Knopman, D.S., Kwok, J.B., Lambert, J.-C., Lee, T., Li, G., Li, S.-C., Loitfelder, M., Lopez, O.L., Lundervold, A.J., Lundqvist, A., Mather, K.A., Mirza, S.S., Nyberg, L., Oostra, B.A., Palotie, A., Papenberg, G., Pattie, A., Petrovic, K., Polasek, O., Psaty, B.M., Redmond, P., Reppermund, S., Rotter, J.I., Schmidt, H., Schuur, M., Schofield, P.W., Scott, R.J., Steen, V.M., Stott, D.J., van Swieten, J.C., Taylor, K.D., Trollor, J., Trompet, S., Uitterlinden, A.G., Weinstein, G., Widen, E., Windham, B.G., Jukema, J.W., Wright, A.F., Wright, M.J., Yang, Q., Amieva, H., Attia, J.R., Bennett, D.A., Brodaty, H., de Craen, A.J.M., Hayward, C., Ikram, M.A., Lindenberger, U., Nilsson, L.-G., Porteous, D.J., Raikkonen, K., Reinvang, I., Rudan, I., Sachdev, P.S., Schmidt, R., Schofield, P.R., Srikanth, V., Starr, J.M., Turner, S.T., Weir, D.R., Wilson, J.F., van Duijn, C., Launer, L., Fitzpatrick, A.L., Seshadri, S., Mosley, T.H.J., Deary, I.J., 2015. Genetic contributions to variation in general cognitive function: a meta-analysis of genome-wide association studies in the CHARGE consortium (N=53949). Mol. Psychiatry 20, 183–192. https://doi.org/https://dx.doi.org/10.1038/mp.2014.188

de Almondes, K.M., Costa, M.V., Malloy-Diniz, L.F., Diniz, B.S., 2016. Insomnia and risk of dementia in older adults: Systematic review and meta-analysis. J. Psychiatr. Res. 77, 109–115. https://doi.org/https://dx.doi.org/10.1016/j.jpsychires.2016.02.021

de Assis, G.G., de Almondes, K.M., 2017. Exercise-dependent BDNF as a modulatory factor for the executive processing of individuals in course of cognitive decline. A systematic review. Front. Psychol. 8.

de Keijzer, C., Gascon, M., Nieuwenhuijsen, M.J., Dadvand, P., 2016. Long-Term Green Space Exposure and Cognition Across the Life Course: a Systematic Review. Curr. Environ. Heal. reports 3, 468–477. https://doi.org/https://dx.doi.org/10.1007/s40572-016-0116-x

Deckers, K., Boxtel, M.P.J., Schiepers, O.J.G., Vugt, M., Sanchez, J.L.M., Anstey, K.J., Brayne, C., Dartigues, J.-F., Engedal, K., Kivipelto, M., Ritchie, K., Starr, J.M., Yaffe, K., Irving, K., Verhey, F.R.J., Kohler, S., 2014. Target risk factors for dementia prevention: A systematic review and Delphi consensus study on the evidence from observational studies. Int. J. Geriatr. Psychiatry 30, 234–246. https://doi.org/http://dx.doi.org/10.1002/gps.4245

Deckers, K., Camerino, I., van Boxtel, M.P.J., Verhey, F.R.J., Irving, K., Brayne, C., Kivipelto, M., Starr, J.M., Yaffe, K., de Leeuw, P.W., Kohler, S., 2017. Dementia risk in renal dysfunction: A systematic review and meta-analysis of prospective studies. Neurology, [Comment in: Neurology. 2017 Nov 21;89(21):2214; PMID: 29158300 [https://www.ncbi.nlm.nih.gov/pubmed/29158300]][Comment in: Neurology. 2017 Nov 21;89(21):2214-2215; PMID: 29158301

[https://www.ncbi.nlm.nih.gov/pubmed/29158301]] 88, 198–208. https://doi.org/https://dx.doi.org/10.1212/WNL.0000000000003482

Deligkaris, P., Panagopoulou, E., Montgomery, A.J., Masoura, E., 2014. Job burnout and cognitive functioning: A systematic review. Work Stress 28, 107–123. https://doi.org/10.1080/02678373.2014.909545

Desai, R., John, A., Stott, J., & Charlesworth, G., 2020. Living alone and risk of dementia: A systematic review and meta-analysis. Ageing Research Reviews, 62, 101122.

Di Marco, L.Y., Marzo, A., Mu~noz-Ruiz, M., Ikram, M.A., Kivipelto, M., Ruefenacht, D., Venneri, A., Soininen, H., Wanke, I., Ventikos, Y.A., Frangi, A.F., 2014. Modifiable lifestyle factors in dementia: A systematic review of longitudinal observational cohort studies. J. Alzheimer's Dis. 42, 119–135.

Dimakakou, E., Johnston, H.J., Streftaris, G., Cherrie, J.W., 2018. Exposure to Environmental and Occupational Particulate Air Pollution as a Potential Contributor to Neurodegeneration and Diabetes: A Systematic Review of Epidemiological Research. Int. J. Environ. Res. Public Health 15. https://doi.org/https://dx.doi.org/10.3390/ijerph15081704

Diniz, B.S., Butters, M.A., Albert, S.M., Dew, M.A., Reynolds, C.F. 3rd, 2013. Late-life depression and risk of vascular dementia and Alzheimer's disease: systematic review and meta-analysis of community-based cohort studies. Br. J. Psychiatry 202, 329–335. https://doi.org/https://dx.doi.org/10.1192/bjp.bp.112.118307

Diniz, B.S., Teixeira, A.L., Cao, F., Gildengers, A., Soares, J.C., Butters, M.A., Reynolds, C.F. 3rd, 2017. History of Bipolar Disorder and the Risk of Dementia: A Systematic Review and Meta-Analysis. Am. J. Geriatr. Psychiatry 25, 357–362. https://doi.org/https://dx.doi.org/10.1016/j.jagp.2016.11.014

Dobbels, B., Peetermans, O., Boon, B., Mertens, G., P, V. de H., V, V.R., 2019. Impact of Bilateral Vestibulopathy on Spatial and Nonspatial Cognition: A Systematic Review. Ear Hear. 40, 757–765. https://doi.org/10.1097/AUD.0000000000000679

Dong, X., Zhang, L., Meng, Q., Gao, Q., 2017. Association Between Interleukin-1A, Interleukin-1B, and Bridging integrator 1 Polymorphisms and Alzheimer's Disease: a standard and Cumulative Meta-analysis. Mol. Neurobiol. 54, 736–747. https://doi.org/https://dx.doi.org/10.1007/s12035-015-9683-3

Dong, Y., Chen, X., Liu, Y., Shu, Y., Chen, T., Xu, L., Li, M., Guan, X., 2018. Do low-serum vitamin E levels increase the risk of Alzheimer disease in older people? Evidence from a meta-analysis of case-control studies. Int. J. Geriatr. Psychiatry 33, e257–e263. https://doi.org/https://dx.doi.org/10.1002/gps.4780

Du, K., Liu, M., Pan, Y., Zhong, X., Wei, M., 2017. Association of Serum Manganese Levels with Alzheimer's Disease and Mild Cognitive Impairment: A Systematic Review and Meta-Analysis. Nutrients 9. https://doi.org/https://dx.doi.org/10.3390/nu9030231

Du, N., Xu, D., Hou, X., Song, X., Liu, C., Chen, Y., Wang, Y., Li, X., 2016. Inverse Association Between Serum Uric Acid Levels and Alzheimer's Disease Risk. Mol. Neurobiol. 53, 2594–2599. https://doi.org/https://dx.doi.org/10.1007/s12035-015-9271-6

Dyer, A., Briggs, R., Mockler, D., Gibney, J., SP, K., 2019. Non-Pharmacological Interventions for Cognition in Patients with Type 2 Diabetes Mellitus: A Systematic Review. QJM. https://doi.org/10.1093/qjmed/hcz053

Elias-Sonnenschein, L.S., Viechtbauer, W., Ramakers, I.H.G.B., Verhey, F.R.J., Visser, P.J., LS, E.-S., Viechtbauer, W., IH, R., FR, V., PJ, V., Elias-Sonnenschein, L.S., Viechtbauer, W., Ramakers, I.H.G.B., Verhey, F.R.J., Visser, P.J., LS, E.-S., Viechtbauer, W., IH, R., FR, V., PJ, V., 2011. Predictive value of APOE-epsilon4 allele for progression from MCI to AD-type dementia: a meta-analysis. J. Neurol. Neurosurg. Psychiatry 82, 1149–1156. https://doi.org/10.1136/jnnp.2010.231555

Ennis, N., Roy, S., Topolovec-Vranic, J., 2015. Memory impairment among people who are homeless: a systematic review. Memory 23, 695–713. https://doi.org/https://dx.doi.org/10.1080/09658211.2014.921714

Etgen, T., Chonchol, M., Forstl, H., Sander, D., 2012a. Chronic kidney disease and cognitive impairment: a systematic review and meta-analysis. Am. J. Nephrol. 35, 474–482. https://doi.org/https://dx.doi.org/10.1159/000338135

Etgen, T., Sander, D., Bickel, H., Sander, K., Forstl, H., 2012b. Vitamin D deficiency, cognitive impairment and dementia: A systematic review and meta-analysis. Dement. Geriatr. Cogn. Disord., Dementia 33, 297–305. https://doi.org/http://dx.doi.org/10.1159/000339702

Fang, W., Jiang, M., Gu, B., Wei, Y., Fan, S., Liao, W., Zheng, Y., Liao, S., Xiong, Y., Li, Y., Xiao, S., Jun, L., 2018. Tooth loss as a risk factor for dementia: Systematic review and meta-analysis of 21 observational studies. BMC Psychiatry 18.

Fei, M., Jianhua, W., 2013. Apolipoprotein ε4-allele as a significant risk factor for conversion from mild cognitive impairment to Alzheimer's disease: a meta-analysis of prospective studies. J. Mol. Neurosci. 50, 257–263. https://doi.org/10.1007/s12031-012-9934-y

Feinkohl, I., Winterer, G., Pischon Tobias; ORCID: http://orcid.org/0000-0003-1568-767X, T.A.I.-O. http://orcid. org/Pischo., 2018. Associations of dyslipidaemia and lipid-lowering treatment with risk of postoperative cognitive dysfunction: A systematic review and meta-analysis. J. Epidemiol. Community Health, British Journal of Preventive & Social Medicine 72, 499–506. https://doi.org/http://dx.doi.org/10.1136/jech-2017-210338

Fink, H.A., Hemmy, L.S., MacDonald, R., Carlyle, M.H., Olson, C.M., Dysken, M.W., McCarten, J.R., Kane, R.L., Garcia, S.A., Rutks, I.R., Ouellette, J., Wilt, T.J., 2015. Intermediate- and Long-Term Cognitive Outcomes After Cardiovascular Procedures in Older Adults: A Systematic Review. Ann. Intern. Med., [Comment in: BMJ. 2015;351:h3877; PMID: 26198722 [https://www.ncbi.nlm.nih.gov/pubmed/26198722]][Comment in: Evid Based Med. 2015 Dec;20(6):221; PMID: 26385489 [https://www.ncbi.nlm.nih.gov/pubmed/26385489]] 163, 107–117. https://doi.org/https://dx.doi.org/10.7326/M14-2793

Forbes, D., Forbes, S.C., Blake, C.M., Thiessen, E.J., Forbes, S., 2015. Exercise programs for people with dementia. Cochrane Database Syst. Rev. https://doi.org/10.1002/14651858.CD006489.pub4

Ford, A.H., GJ, H., BB, Y., Golledge, J., Flicker, L., OP, A., Hankey, G.J., Yeap, B.B., Golledge, J., Flicker, L., Almeida, O.P., AH, F., GJ, H., BB, Y., Golledge, J., Flicker, L., OP, A., 2018. Hearing loss and the risk of dementia in later life. Maturitas 112, 1–11. https://doi.org/10.1016/j.maturitas.2018.03.004

Ford, E., Greenslade, N., Paudyal, P., Bremner, S., Smith, H.E., Banerjee, S., Sadhwani, S., Rooney, P., Oliver, S., Cassell, J., 2018. Predicting dementia from primary care records: A systematic review and meta-analysis. PLoS One 13, e0194735. https://doi.org/https://dx.doi.org/10.1371/journal.pone.0194735

Forero, D.A., Gonzalez-Giraldo, Y., Lopez-Quintero, C., Castro-Vega, L.J., Barreto, G.E., Perry, G., 2016. Meta-analysis of Telomere Length in Alzheimer's Disease. J. Gerontol. A. Biol. Sci. Med. Sci. 71, 1069–1073. https://doi.org/https://dx.doi.org/10.1093/gerona/glw053

Forlenza, O. V, Diniz, B.S., Stella, F., Teixeira, A.L., Gattaz, W.F., 2013. Mild cognitive impairment. Part 1: clinical characteristics and predictors of dementia. Rev. Bras. Psiquiatr. 35, 178–185.

Fotuhi, M., Mohassel, P., Yaffe, K., 2009. Fish consumption, long-chain omega-3 fatty acids and risk of cognitive decline or Alzheimer disease: a complex association. Nat. Clin. Pract. Neurol. 5, 140–152. https://doi.org/https://dx.doi.org/10.1038/ncpneuro1044

Galbiati, A., Verga, L., Giora, E., Zucconi, M., Laura, F.-S., 2019. The risk of neurodegeneration in REM sleep behavior disorder: A systematic review and meta-analysis of longitudinal studies. Sleep Med. Rev. 43, 37–46. https://doi.org/http://dx.doi.org/10.1016/j.smrv.2018.09.008

Gao, L., Zhang, Y., Deng, J., Yu, W., Yu, Y., 2016. Polymorphisms of chat but not TFAM or VR22 are associated with alzheimer disease risk. Med. Sci. Monit. 22, 1924–1935. https://doi.org/10.12659/MSM.895984

Gao, Y., Huang, C., Zhao, K., Ma, L., Qiu, X., Zhang, L., Xiu, Y., Chen, L., Lu, W., Huang, C., Tang, Y., Xiao, Q., 2013. Depression as a risk factor for dementia and mild cognitive impairment: a meta-analysis of longitudinal studies. Int. J. Geriatr. Psychiatry 28, 441–449. https://doi.org/10.1002/gps.3845

Gimson, A., Schlosser, M., Huntley, J.D., Marchant, N.L., 2018. Support for midlife anxiety diagnosis as an independent risk factor for dementia: a systematic review. BMJ Open 8, e019399. https://doi.org/https://dx.doi.org/10.1136/bmjopen-2017-019399

Goodwill, A.M., Cassandra, S., 2017. A Systematic Review and Meta-Analysis of The Effect of Low Vitamin D on Cognition. J. Am. Geriatr. Soc. 65, 2161–2168. https://doi.org/10.1111/jgs.15012

Gore, R.L., Vardy, E.R.L.C., O'Brien, J.T., 2014. Delirium and dementia with Lewy bodies: Distinct diagnoses or part of the same spectrum? J. Neurol. Neurosurg. Psychiatry, Journal of Neurology & Psychiatry 86, 50–59. https://doi.org/http://dx.doi.org/10.1136/jnnp-2013-306389

Guan, F., Gu, J., Hu, F., Zhu, Y., Wang, W., 2012. Association between α1-antichymotrypsin signal peptide -15A/T polymorphism and the risk of Alzheimer's disease: a meta-analysis. Mol. Biol. Rep. 39, 6661–6669. https://doi.org/https://dx.doi.org/10.1007/s11033-012-1472-8

Guan, J.-W., Huang, C.-Q., Li, Y.-H., Wan, C.-M., You, C., Wang, Z.-R., Liu, Y.-Y., Liu, Q.-X., 2011. No association between hypertension and risk for Alzheimer's disease: A meta-analysis of longitudinal studies. J. Alzheimer's Dis. 27, 799–807.

Guerchet, M., Aboyans, V., Nubukpo, P., Lacroix, P., Clement, J.-P., Preux, P.-M., 2011. Ankle-brachial index as a marker of cognitive impairment and dementia in general population. A systematic review. Atherosclerosis 216, 251–257. https://doi.org/https://dx.doi.org/10.1016/j.atherosclerosis.2011.03.024

Gulpers, B., Ramakers, I., Hamel, R., Kohler, S., Oude Voshaar, R., Verhey, F., 2016. Anxiety as a Predictor for Cognitive Decline and Dementia: A Systematic Review and Meta-Analysis. Am. J. Geriatr. Psychiatry, [Comment in: Am J Geriatr Psychiatry. 2016 Oct;24(10):843-5; PMID: 27591156 [https://www.ncbi.nlm.nih.gov/pubmed/27591156]] 24, 823–842. https://doi.org/https://dx.doi.org/10.1016/j.jagp.2016.05.015

Lenart-Bugla et al., 2022: What do we know about social and non-social factors influencing the pathway from cognitive health to dementia? A systematic review of reviews.

Gunnarsson, L.-G., Bodin, L., 2019. Occupational Exposures and Neurodegenerative Diseases-A Systematic Literature Review and Meta-Analyses. Int. J. Environ. Res. Public Health 16. https://doi.org/https://dx.doi.org/10.3390/ijerph16030337

Guure, C.B., Ibrahim, N.A., Adam, M.B., Said, S.M., 2017. Impact of Physical Activity on Cognitive Decline, Dementia, and Its Subtypes: Meta-Analysis of Prospective Studies. Biomed Res. Int. 2017, 9016924. https://doi.org/https://dx.doi.org/10.1155/2017/9016924

Haaksma, M.L., Vilela, L.R., Marengoni, A., Calderon-Larranaga, A., Leoutsakos, J.-M.S., Olde Rikkert, M.G.M., Melis, R.J.F., 2017. Comorbidity and progression of late onset Alzheimer's disease: A systematic review. PLoS One 12, e0177044. https://doi.org/https://dx.doi.org/10.1371/journal.pone.0177044

Hagg, S., Zhan, Y., Karlsson, R., Gerritsen, L., Ploner, A., van der Lee, S.J., Broer, L., Deelen, J., Marioni, R.E., Wong, A., Lundquist, A., Zhu, G., Hansell, N.K., Sillanpaa, E., Fedko, I.O., Amin, N.A., Beekman, M., de Craen, A.J.M., Degerman, S., Harris, S.E., Kan, K.-J., Martin-Ruiz, C.M., Montgomery, G.W., Group, N.C.W., Adolfsson, A.N., Reynolds, C.A., Samani, N.J., Suchiman, H.E.D., Viljanen, A., von Zglinicki, T., Wright, M.J., Hottenga, J.-J., Boomsma, D.I., Rantanen, T., Kaprio, J.A., Nyholt, D.R., Martin, N.G., Nyberg, L., Adolfsson, R., Kuh, D., Starr, J.M., Deary, I.J., Slagboom, P.E., van Duijn, C.M., Codd, V., Pedersen, N.L., 2017. Short telomere length is associated with impaired cognitive performance in European ancestry cohorts. Transl. Psychiatry 7, e1100. https://doi.org/https://dx.doi.org/10.1038/tp.2017.73

Hajduk, A.M., Kiefe, C.I., Person, S.D., Gore, J.G., Saczynski, J.S., 2013. Cognitive change in heart failure: a systematic review. Circ. Cardiovasc. Qual. Outcomes 6, 451–460. https://doi.org/https://dx.doi.org/10.1161/CIRCOUTCOMES.113.000121

Hamer, M., Chida, M., 2009. Physical activity and risk of neurodegenerative disease: A systematic review of prospective evidence. Psychol. Med. 39, 3–11. https://doi.org/http://dx.doi.org/10.1017/S0033291708003681

Hao, Z., Wu, B., Wang, D., Liu, D., 2011. Association between metabolic syndrome and cognitive decline: A systematic review of prospective population-based studies. Acta Neuropsychiatr. 23, 69–74. https://doi.org/http://dx.doi.org/10.1111/j.1601-5215.2011.00527.x

Hasselbalch, B.J., Knorr, U., Kessing, L.V., 2011. Cognitive impairment in the remitted state of unipolar depressive disorder: A systematic review. J. Affect. Disord. 134, 20–31. https://doi.org/10.1016/j.jad.2010.11.011

He, D., Lu, W., Chang, K., Liu, Y., Zhang, J., Zeng, Z., 2013. Vascular endothelial growth factor polymorphisms and risk of Alzheimer's disease: a meta-analysis. Gene 518, 296–302. https://doi.org/https://dx.doi.org/10.1016/j.gene.2013.01.021

Henneghan, A., 2016. Modifiable factors and cognitive dysfunction in breast cancer survivors: a mixed-method systematic review. Support. Care Cancer 24, 481–497. https://doi.org/https://dx.doi.org/10.1007/s00520-015-2927-y

Heringa, S.M., Bouvy, W.H., van den Berg, E., Moll, A.C., Kappelle, L.J., Biessels, G.J., 2013. Associations between retinal microvascular changes and dementia, cognitive functioning, and brain imaging abnormalities: a systematic review. J. Cereb. Blood Flow Metab. 33, 983–995. https://doi.org/https://dx.doi.org/10.1038/jcbfm.2013.58

Hersi, M., Irvine, B., Gupta, P., Gomes, J., Birkett, N., Krewski, D., 2017. Risk factors associated with the onset and progression of Alzheimer's disease: A systematic review of the evidence. Neurotoxicology 61, 143–187. https://doi.org/https://dx.doi.org/10.1016/j.neuro.2017.03.006

Hill, E., Goodwill, A.M., Gorelik, A., Edward, S., 2019. Diet and biomarkers of Alzheimer's disease: a systematic review and meta-analysis. Neurobiol. Aging 76, 45–52. https://doi.org/http://dx.doi.org/10.1016/j.neurobiolaging.2018.12.008

Hu, C., Yu, D., Sun, X., Zhang, M., Wang, L., Qin, H., 2017. The prevalence and progression of mild cognitive impairment among clinic and community populations: a systematic review and meta-analysis. Int. psychogeriatrics 29, 1595–1608. https://doi.org/https://dx.doi.org/10.1017/S1041610217000473

Hua, Y., Zhao, H., Kong, Y., Lu, X., 2012. Meta-analysis of the association between the interleukin-1A-889C/T polymorphism and Alzheimer's disease. J. Neurosci. Res. 90, 1681–1692. https://doi.org/http://dx.doi.org/10.1002/jnr.23062

Hua, Y., Zhao, H., Kong, Y., Ye, M., 2011. Association between the MTHFR gene and Alzheimer's disease: a meta-analysis. Int. J. Neurosci. 121, 462–471. https://doi.org/https://dx.doi.org/10.3109/00207454.2011.578778

Huang, R., Tian, S., Cai, R., Sun, J., Xia, W., Dong, X., Shen, Y., Wang, S., 2017. Saitohin Q7R polymorphism is associated with late-onset Alzheimer's disease susceptibility among caucasian populations: a meta-analysis. J. Cell. Mol. Med. 21, 1448–1456. https://doi.org/https://dx.doi.org/10.1111/jcmm.13079

Huckans, M., Hutson, L., Twamley, E., Jak, A., Kaye, J., Jeffrey, S., 2013. Efficacy of cognitive rehabilitation therapies for mild cognitive impairment (MCI) in older adults: Working toward a theoretical model and evidence-based interventions. Neuropsychol. Rev. 23, 63–80. https://doi.org/http://dx.doi.org/10.1007/s11065-013-9230-9

Hui, D.S., Morley, J.E., Mikolajczak, P.C., Lee, R., 2015. Atrial fibrillation: A major risk factor for cognitive decline. Am. Heart J. 169, 448–456. https://doi.org/https://dx.doi.org/10.1016/j.ahj.2014.12.015

Iizuka, A., Suzuki, H., Ogawa, S., KE, K.-C., Kobayashi, M., Takebayashi, T., Fujiwara, Y., 2019. Can cognitive leisure activity prevent cognitive decline in older adults? A systematic review of intervention studies. Geriatr. Gerontol. Int. 19, 469–482. https://doi.org/10.1111/ggi.13671

Jalilian, H., SH, T., Röösli, M., Neghab, M., 2018. Occupational exposure to extremely low frequency magnetic field and risk of Alzheimer disease: A systematic review and meta-analysis. Neurotoxicology 69, 242–252. https://doi.org/10.1016/j.neuro.2017.12.005

Jia, R., Liang, J., Xu, Y., Wang, Y., 2019. Effects of physical activity and exercise on the cognitive function of patients with Alzheimer disease: a meta-analysis. BMC Geriatr. 19, 181. https://doi.org/10.1186/s12877-019-1175-2

Jiang, Y., Zhu, Z., Shi, J., An, Y., Zhang, K., Wang, Y., Li, S., Jin, L., Ye, W., Cui, M., Chen, X., 2019. Metabolomics in the Development and Progression of Dementia: A Systematic Review. Front. Neurosci. 13, 343. https://doi.org/https://dx.doi.org/10.3389/fnins.2019.00343

Jin, C., Li, W., Yuan, J., Xu, W., Cheng, Z., 2012. Association of the CR1 polymorphism with late-onset Alzheimer's disease in Chinese Han populations: a meta-analysis. Neurosci. Lett. 527, 46–49. https://doi.org/https://dx.doi.org/10.1016/j.neulet.2012.08.032

Julien, J., Joubert, S., Ferland, M.-C., Frenette, L.C., Boudreau-Duhaime, M.M., Malo-Veronneau, L., de Guise, E., 2017. Association of traumatic brain injury and Alzheimer disease onset: A systematic review. Ann. Phys. Rehabil. Med. 60, 347–356. https://doi.org/https://dx.doi.org/10.1016/j.rehab.2017.03.009

Jun, G., Naj, A.C., Beecham, G.W., Wang, L.-S., Buros, J., Gallins, P.J., Buxbaum, J.D., Ertekin-Taner, N., Fallin, M.D., Friedland, R., Inzelberg, R., Kramer, P., Rogaeva, E., St. George-Hyslop, P., Cantwell, L.B., Dombroski, B.A., Saykin, A.J., Reiman, E.M., Bennett, D.A., Morris, J.C., Lunetta, K.L., Martin, E.R., Montine, T.J., Goate, A.M., Blacker, D., Tsuang, D.W., Beekly, D., Cupples, L.A., Hakonarson, H., Kukull, W., Foroud, T.M., Haines, J., Mayeux, R., Farrer, L.A., Pericak-Vance, M.A., Gyungah, S., 2010. Meta-analysis confirms CR1, CLU, and PICALM as Alzheimer disease risk loci and reveals interactions with APOE genotypes. Arch. Neurol., A.M.A. Archives of Neurology, JAMA Neurology 67, 1473–1484. https://doi.org/http://dx.doi.org/10.1001/archneurol.2010.201

Keage, H.A.D., Carare, R.O., Friedland, R.P., Ince, P.G., Love, S., Nicoll, J.A., Wharton, S.B., Weller, R.O., Brayne, C., 2009. Population studies of sporadic cerebral amyloid angiopathy and dementia: a systematic review. BMC Neurol. 9, 3. https://doi.org/https://dx.doi.org/10.1186/1471-2377-9-3

Kelly, M.E., Duff, H., Kelly, S., McHugh Power, J.E., Brennan, S., Lawlor, B.A., Loughrey, D.G., 2017. The impact ofsocial activities, social networks, social support and social relationships on the cognitive functioning of healthy older adults: A systematic review SR. Syst. Rev. 6. https://doi.org/10.1186/s13643-017-0632-2

Killin, L.O.J., Starr, J.M., Shiue, I.J., Russ, T.C., 2016. Environmental risk factors for dementia: a systematic review. BMC Geriatr. 16, 175.

Kivimäki, M., Singh-Manoux, A., Pentti, J., Sabia, S., ST, N., Alfredsson, L., Goldberg, M., Knutsson, A., Koskenvuo, M., Koskinen, A., Kouvonen, A., Nordin, M., Oksanen, T., Strandberg, T., SB, S., Theorell, T., Vahtera, J., Väänänen, A., Virtanen, M., Westerholm, P., Westerlund, H., Zins, M., Seshadri, S., GD, B., PN, S., MJ, S., JV, L., JE, F., Jokela, M., consortium, I.-W., 2019. Physical inactivity, cardiometabolic disease, and risk of dementia: an individual-participant meta-analysis. BMJ 365, l1495. https://doi.org/10.1136/bmj.l1495

Klimova, B., Kuca, K., Maresova, P., 2018. Global view on Alzheimer's disease and diabetes mellitus: Threats, risks and treatment Alzheimer's disease and diabetes mellitus. Curr. Alzheimer Res. 15, 1277–1282. https://doi.org/http://dx.doi.org/10.2174/1567205015666180925110222

Kobayashi-Cuya, K.E., Sakurai, R., Suzuki, H., Ogawa, S., Takebayashi, T., Fujiwara, Y., 2018. Observational Evidence of the Association Between Handgrip Strength, Hand Dexterity, and Cognitive Performance in Community-Dwelling Older Adults: A Systematic Review. J. Epidemiol. 28, 373–381. https://doi.org/https://dx.doi.org/10.2188/jea.JE20170041

Kopf, D., Frölich, L., 2009. Risk of Incident Alzheimer's Disease in Diabetic Patients: A Systematic Review of Prospective Trials. J. Alzheimer's Dis. 16, 677–685. https://doi.org/10.3233/JAD-2009-1011

Koyama, A., O'Brien, J., Weuve, J., Blacker, D., Metti, A.L., Yaffe, K., 2013. The role of peripheral inflammatory markers in dementia and Alzheimer's disease: a meta-analysis. J. Gerontol. A. Biol. Sci. Med. Sci. 68, 433–440. https://doi.org/https://dx.doi.org/10.1093/gerona/gls187

Koyama, A., Okereke, O.I., Yang, T., Blacker, D., Selkoe, D.J., Grodstein, F., 2012. Plasma amyloid-beta as a predictor of dementia and cognitive decline: A systematic review and meta-analysis. Arch. Neurol., A.M.A. Archives of Neurology, JAMA Neurology 69, 824–831. https://doi.org/http://dx.doi.org/10.1001/archneurol.2011.1841

Krishna, M., Jones, S., Maden, M., Du, B., Mc, R., Kumaran, K., SC, K., CHD, F., 2019. Size at birth and cognitive ability in late life: A systematic review. Int. J. Geriatr. Psychiatry 34, 1139–1169. https://doi.org/10.1002/gps.5138

Kueper, J.K., Speechley, M., Lingum, N.R., Montero-Odasso, M., 2017. Motor function and incident dementia: a systematic review and meta-analysis. Age Ageing, [Comment in: Age Ageing. 2017 Sep 1;46(5):704-705; PMID: 28633430 [https://www.ncbi.nlm.nih.gov/pubmed/28633430]] 46, 729–738. https://doi.org/https://dx.doi.org/10.1093/ageing/afx084

Kuiper, J.S., Zuidersma, M., Oude Voshaar, R.C., Zuidema, S.U., van den Heuvel, E.R., Stolk, R.P., Smidt, N., 2015. Social relationships and risk of dementia: A systematic review and meta-analysis of longitudinal cohort studies. Ageing Res. Rev. 22, 39–57. https://doi.org/https://dx.doi.org/10.1016/j.arr.2015.04.006

Kuiper, J.S., Zuidersma, M., Zuidema, S.U., Burgerhof, J.G.M., Stolk, R.P., Oude Voshaar, R.C., Smidt, N., 2016. Social relationships and cognitive decline: a systematic review and meta-analysis of longitudinal cohort studies. Int. J. Epidemiol. 45, 1169–1206. https://doi.org/10.1093/ije/dyw089

Kunkle, B., Grenier-Boley, B., Sims, R., JC, B., Damotte, V., AC, N., Boland, A., Vronskaya, M., SJ, van der L., Amlie-Wolf, A., Bellenguez, C., Frizatti, A., Chouraki, V., ER, M., Sleegers, K., Badarinarayan, N., Jakobsdottir, J., KL, H.-N., Moreno-Grau, S., Olaso, R., Raybould, R., Chen, Y., AB, K., Hiltunen, M., Morgan, T., Ahmad, S., BN, V., Epelbaum, J., Hoffmann, P., Boada, M., GW, B., JG, G., Harold, D., AL, F., Valladares, O., ML, M., Gerrish, A., AV, S., Qu, L., Bacq, D., Denning, N., Jian, X., Zhao, Y., M, D.Z., NC, F., SH, C., Mateo, I., JT, H., HH, A., Malamon, J., Sanchez-Garcia, F., Patel, Y., JA, B., BA, D., MCD, N., Daniilidou, M., Eiriksdottir, G., Mukherjee, S., Wallon, D., Uphill, J., Aspelund, T., LB, C., Garzia, F., Galimberti, D., Hofer, E., Butkiewicz, M., Fin, B., Scarpini, E., Sarnowski, C., WS, B., Meslage, S., Kornhuber, J., CC, W., Song, Y., RC, B., Engelborghs, S., Sordon, S., Voijnovic, D., PM, A., Vandenberghe, R., Mayhaus, M., LA, C., MS, A., PP, D.D., Gu, W., JJ, H., Beekly, D., Squassina, A., AM, H., Orellana, A., Blacker, D., Rodriguez-Rodriguez, E., Lovestone, S., ME, G., RS, D., Munoz-Fernadez, C., Sussams, R., Lin, H., TJ, F., YA, B., Holmes, C., Karamujić-Čomić, H., MP, F., Thonberg, H., Maier, W., Roschupkin, G., Ghetti, B., Giedraitis, V., Kawalia, A., Li, S., RM, H., Kilander, L., Moebus, S., Hernández, I., MI, K., Brundin, R., Turton, J., Yang, Q., MJ, K., Concari, L., Lord, J., AS, B., CD, K., Helisalmi, S., Kloszewska, I., WA, K., AM, K., Lynch, A., Tarraga, L., EB, L., Haapasalo, A., Lawlor, B., TH, M., RB, L., Solfrizzi, V., Gill, M., WT, L., TJ, M., Frisardi, V., Diez-Fairen, M., Rivadeneira, F., RC, P., Deramecourt, V., Alvarez, I., Salani, F., Ciaramella, A., Boerwinkle, E., EM, R., Fievet, N., JI, R., JS, R., Hanon, O., Cupidi, C., AG, A.U., DR, R., Dufouil, C., RG, M., I, de R., Sano, M., Brice, A., Cecchetti, R., PS, G.-H., Ritchie, K., Tsolaki, M., DW, T., Dubois, B., Craig, D., CK, W., Soininen, H., Avramidou, D., RL, A., Fratiglioni, L., Germanou, A., LG, A., Keller, L., Koutroumani, M., SE, A., Panza, F., Gkatzima, O., Asthana, S., Hannequin, D., Whitehead, P., CS, A., Caffarra, P., Hampel, H., Quintela, I., Carracedo, Á., Lannfelt, L., DC, R., LL, B., Pasquier, F., Frölich, L., Barral, S., McGuinness, B., TG, B., JA, J., JT, B., Passmore, P., EH, B., JM, S., TD, B., JD, W., BF, B., MK, L., JD, Bowen, Proitsi, P., Boxer, A., JF, P., JR, B., JSK, K., JM, B., Mancuso, M., JD, Buxbaum, Bonuccelli, U., NJ, C., McQuillin, A., Cao, C., Livingston, G., CS, C., NJ, B., CM, C., Hardy, J., RM, C., Bras, J., MM, C., Guerreiro, R., Allen, M., HC, C., Fisher, E., Masullo, C., EA, C., DeCarli, C., Bisceglio, G., Dick, M., Ma, L., Duara, R., NR, G.-R., DA, E., Hodges, A., KM, F., Scherer, M., KB, F., Riemenschneider, M., DW, F., Heun, R., MR, F., Kölsch, H., Ferris, S., Leber, M., TM, F., Heuser, I., DR, G., Giegling, I., Gearing, M., Hüll, M., DH, G., JR, G., Morris, J., RC, G., Mayo, K., JH, G., Feulner, T., RL, H., LE, H., Drichel, D., LS, H., TD, C., MJ, H., Hollingworth, P., CM, H., BT, H., Marshall, R., GP, J., Meggy, A., Abner, E., GE, M., LW, J., Leonenko, G., LM, R., GR, J., CT, B., Grozeva, D., Karydas, A., Russo, G., JA, K., Kim, R., Jessen, F., NW, K., Vellas, B., JH, K., Vardy, E., FM, L., KH, J., JJ, L., Dichgans, M., JB, L., Mann,

D., AI, L., Pickering-Brown, S., AP, L., Klopp, N., KL, L., HE, W., CG, L., Morgan, K., DC, Marson, Brown, K., Martiniuk, F., Medway, C., DC, Mash, MM, N., Masliah, E., NM, H., WC, M., Daniele, A., SM, M., Bayer, A., AN, M., Gallacher, J., AC, M., H, van den B., Mesulam, M., Brayne, C., BL, M., Riedel-Heller, S., CA, M., JW, M., Al-Chalabi, A., JC, M., CE, S., AJ, M., Wiltfang, J., O'Bryant, S., JM, O., Alvarez, V., JE, P., AB, S., HL, P., Collinge, J., WR, P., Mead, S., Peskind, E., DH, C., Rossor, M., Pierce, A., NS, R., WW, P., Nacmias, B., Potter, H., Sorbi, S., JF, Q., Sacchinelli, E., Raj, A., Spalletta, G., Raskind, M., Caltagirone, C., Bossù, P., MD, O., Reisberg, B., Clarke, R., Reitz, C., AD, S., JM, R., Warden, D., ED, R., Wilcock, G., Rogaeva, E., AC, B., HJ, R., Gallo, M., RN, R., Ben-Shlomo, Y., MA, S., Mecocci, P., AJ, S., Pastor, P., ML, C., JM, V., JA, Schneider, LS, S., Slifer, S., WW, S., AG, S., JA, Sonnen, Spina, S., RA, S., RH, S., Tang, M., RE, T., JQ, T., JC, T., VM, V.D., LJ, V.E., HV, V., JP, V., Weintraub, S., KA, W.-B., KC, W., Williamson, J., TS, W., RL, W., CB, W., CE, Y., Yu, L., Saba, Y., Alzheimer Disease Genetics Consortium (ADGC), European Alzheimer's Disease Initiative (EADI), Cohorts for Heart and Aging Research in Genomic Epidemiology Consortium (CHARGE), Genetic and Environmental Risk in AD/Defining Genetic, P. and E.R. for A.D.C. (GERAD/PERADES), Pilotto, A., MJ, B., Peters, O., PK, C., Bennett, D., Bosco, P., Coto, E., Boccardi, V., PL, D.J., Lleo, A., Warner, N., OL, L., Ingelsson, M., Deloukas, P., Cruchaga, C., Graff, C., Gwilliam, R., Fornage, M., AM, G., Sanchez-Juan, P., PG, K., Amin, N., Ertekin-Taner, N., Berr, C., Debette, S., Love, S., LJ, L., SG, Y., JF, Dartigues, Corcoran, C., MA, I., DW, D., Nicolas, G., Campion, D., Tschanz, J., Schmidt, H., Hakonarson, H., Clarimon, J., Munger, R., Schmidt, R., LA, F., C, V.B., O'Donovan, C.M., AL, D., Jones, L., JL, H., JF, Deleuze, MJ, O., Gudnason, V., Mayeux, R., Escott-Price, V., BM, P., Ramirez, A., LS, W., Ruiz, A., CM, van D., PA, H., Seshadri, S., Williams, J., Amouyel, P., GD, S., JC, L., MA, P.-V., 2019. Genetic meta-analysis of diagnosed Alzheimer's disease identifies new risk loci and implicates Aβ, tau, immunity and lipid processing. Nat. Genet. 51, 414–430. https://doi.org/10.1038/s41588-019-0358-2

Kuzma, E., Hannon, E., Zhou, A., Lourida, I., Bethel, A., Levine, D.A., Lunnon, K., Thompson-Coon, J., Hypponen, E., Llewellyn, D.J., 2018. Which risk factors causally influence dementia? A systematic review of Mendelian randomization studies. J. Alzheimer's Dis. 64, 181–193. https://doi.org/http://dx.doi.org/10.3233/JAD-180013

Kwok, C.S., Loke, Y.K., Hale, R., Potter, J.F., S., M.C., 2011. Atrial fibrillation and incidence of dementia: A systematic review and meta-analysis. Neurology 76, 914–922. https://doi.org/http://dx.doi.org/10.1212/WNL.0b013e31820f2e38

Lafortune, L., Martin, S., Kelly, S., Kuhn, I., Remes, O., Cowan, A., Brayne, C., 2016. Behavioural Risk Factors in Mid-Life Associated with Successful Ageing, Disability, Dementia and Frailty in Later Life: A Rapid Systematic Review. PLoS One 11, e0144405. https://doi.org/https://dx.doi.org/10.1371/journal.pone.0144405

Lai, Y.C., Yew, Y.W., Lambert, W.C., 2016. Bullous pemphigoid and its association with neurological diseases: a systematic review and meta-analysis. J. Eur. Acad. Dermatol. Venereol. 30, 2007–2015. https://doi.org/https://dx.doi.org/10.1111/jdv.13660

Lam, E.W.K., Chung, F., Wong, J., 2017. Sleep-Disordered Breathing, Postoperative Delirium, and Cognitive Impairment. Anesth. Analg., [Comment in: Anesth Analg. 2018 Jan;126(1):368-369; PMID: 29189268 [https://www.ncbi.nlm.nih.gov/pubmed/29189268]][Comment in: Anesth Analg. 2018 Jan;126(1):369-370; PMID: 29189272 [https://www.ncbi.nlm.nih.gov/pubmed/29189272]] 124, 1626–1635. https://doi.org/https://dx.doi.org/10.1213/ANE.0000000000001914

Lancaster, C., Tabet, N., Rusted, J., 2017. The elusive nature of APOE epsilon4 in mid-adulthood: Understanding the cognitive profile. J. Int. Neuropsychol. Soc. 23, 239–253. https://doi.org/http://dx.doi.org/10.1017/S1355617716000990

Lenart-Bugla et al., 2022: What do we know about social and non-social factors influencing the pathway from cognitive health to dementia? A systematic review of reviews.

Last, N., Tufts, E., E., A.L., 2017. The effects of meditation on grey matter atrophy and neurodegeneration: A systematic review. J. Alzheimer's Dis. 56, 275–286. https://doi.org/http://dx.doi.org/10.3233/JAD-160899

Lee, J., 2018. The Relationship Between Physical Activity and Dementia: A Systematic Review and Meta-Analysis of Prospective Cohort Studies. J. Gerontol. Nurs. 44, 22–29. https://doi.org/https://dx.doi.org/10.3928/00989134-20180814-01

Lee, Y., Back, J.H., Kim, J., Kim, S.-H., Na, D.L., Cheong, H.-K., Hong, C.H., Kim Yunhwan; ORCID: http://orcid.org/0000-0001-8484-4750, Cheong, Hae-Kwan; ORCID: http://orcid.org/0000-0003-2758-9399, Y.G.A.I.-O. http://orcid. org/Le., 2010. Systematic review of health behavioral risks and cognitive health in older adults. Int. Psychogeriatrics 22, 174–187. https://doi.org/http://dx.doi.org/10.1017/S1041610209991189

Lehert, P., Villaseca, P., Hogervorst, E., Maki, P.M., Henderson, V.W., 2015. Individually modifiable risk factors to ameliorate cognitive aging: a systematic review and meta-analysis. Climacteric 18, 678–689. https://doi.org/https://dx.doi.org/10.3109/13697137.2015.1078106

Lekoubou, A., Echouffo-Tcheugui, J.B., Kengne, A.P., 2014. Epidemiology of neurodegenerative diseases in sub-Saharan Africa: a systematic review. BMC Public Health 14, 653. https://doi.org/https://dx.doi.org/10.1186/1471-2458-14-653

Leng, Y., McEvoy, C.T., Allen, I.E., Yaffe, K., 2017. Association of Sleep-Disordered Breathing With Cognitive Function and Risk of Cognitive Impairment: A Systematic Review and Meta-analysis. JAMA Neurol., [Erratum in: JAMA Neurol. 2018 Jan 1;75(1):133; PMID: 29159415 [https://www.ncbi.nlm.nih.gov/pubmed/29159415]] 74, 1237–1245. https://doi.org/https://dx.doi.org/10.1001/jamaneurol.2017.2180

Lennon, M., Makkar, S., Crawford, J., Sachdev, P., 2019. Midlife Hypertension and Alzheimer's Disease: A Systematic Review and Meta-Analysis. J. Alzheimers. Dis. https://doi.org/10.3233/JAD-190474

Li, J.-Q., Tan, Lan, Wang, H.-F., Tan, M.-S., Tan, Lin, Xu, W., Zhao, Q.-F., Wang, J., Jiang, T., Yu, J.-T., 2016. Risk factors for predicting progression from mild cognitive impairment to Alzheimer's disease: a systematic review and meta-analysis of cohort studies. J. Neurol. Neurosurg. Psychiatry 87, 476–484. https://doi.org/https://dx.doi.org/10.1136/jnnp-2014-310095

Li, J., Cesari, M., Liu, F., Dong, B., Vellas, B., 2017. Effects of Diabetes Mellitus on Cognitive Decline in Patients with Alzheimer Disease: A Systematic Review. Can. J. diabetes 41, 114–119. https://doi.org/https://dx.doi.org/10.1016/j.jcjd.2016.07.003

Li, L., Fisher, M., Lau, W.-L., Moradi, H., Cheung, A., Thai, G., Handwerker, J., Kalantar-Zadeh, K., 2015. Cerebral microbleeds and cognitive decline in a hemodialysis patient: Case report and review of literature. Hemodial. Int. 19, E1-7. https://doi.org/https://dx.doi.org/10.1111/hdi.12210

Li, M., Wang, W., Li, Y., Wang, L., Shen, X., Tang, Z., 2013. CYP46A1 intron-2T/C polymorphism and Alzheimer's disease: an updated meta-analysis of 16 studies including 3,960 cases and 3,828 controls. Neurosci. Lett. 549, 18–23. https://doi.org/https://dx.doi.org/10.1016/j.neulet.2013.06.011

Li, Y.-Q., Tan, M.-S., Wang, H.-F., Tan, C.-C., Zhang, W., Zheng, Z.-J., Kong, L.-L., Wang, Z.-X., Tan, Lin, Jiang, T., Tan, Lan, Yu, J.-T., 2016. Common variant in PTK2B is associated with late-onset Alzheimer's disease: A replication study and meta-analyses. Neurosci. Lett. 621, 83–87. https://doi.org/https://dx.doi.org/10.1016/j.neulet.2016.04.020

Lenart-Bugla et al., 2022: What do we know about social and non-social factors influencing the pathway from cognitive health to dementia? A systematic review of reviews.

Li, Y., Li, Yongming, Li, X., Zhang, S., Zhao, J., Zhu, X., Tian, G., 2017. Head Injury as a Risk Factor for Dementia and Alzheimer's Disease: A Systematic Review and Meta-Analysis of 32 Observational Studies. PLoS One 12, e0169650. https://doi.org/https://dx.doi.org/10.1371/journal.pone.0169650

Liapis, J., Harding, K.E., 2017. Meaningful use of computers has a potential therapeutic and preventative role in dementia care: A systematic review. Australas. J. Ageing 36, 299–307. https://doi.org/https://dx.doi.org/10.1111/ajag.12446

Lin, M., Zhao, L., Fan, J., Lian, X.-G., Ye, J.-X., Wu, L., Lin, H., 2012. Association between HFE polymorphisms and susceptibility to Alzheimer's disease: a meta-analysis of 22 studies including 4,365 cases and 8,652 controls. Mol. Biol. Rep. 39, 3089–3095. https://doi.org/https://dx.doi.org/10.1007/s11033-011-1072-z

Lin, Y., Cheng, S., Xie, Z., Zhang, D., 2014. Association of rs6265 and rs2030324 polymorphisms in brain-derived neurotrophic factor gene with Alzheimer's disease: a meta-analysis. PLoS One 9, e94961. https://doi.org/https://dx.doi.org/10.1371/journal.pone.0094961

Liu, B., Shen, Y., Cen, L., Tang, Y., 2012. Apolipoprotein E gene polymorphism in a Chinese population with vascular dementia: a meta-analysis. Dement. Geriatr. Cogn. Disord. 33, 96–103. https://doi.org/https://dx.doi.org/10.1159/000337025

Liu, H., Liu, M., Li, W., Wu, B., SH, Z., Fang, Y., Wang, Y., 2009. Association of ACE I/D gene polymorphism with vascular dementia: a meta-analysis. J. Geriatr. Psychiatry Neurol. 22, 10–22. https://doi.org/10.1177/0891988708328221

Liu, H., Yang, M., GM, L., Qiu, Y., Zheng, J., Du, X., JL, W., RW, L., 2010. The MTHFR C677T polymorphism contributes to an increased risk for vascular dementia: a meta-analysis. J. Neurol. Sci. 294, 74–80. https://doi.org/10.1016/j.jns.2010.04.001

Liu, Q.-P., Wu, Y.-F., Cheng, H.-Y., Xia, T., Ding, H., Wang, H., Wang, Z.-M., Xu, Y., 2016. Habitual coffee consumption and risk of cognitive decline/dementia: A systematic review and meta-analysis of prospective cohort studies. Nutrition 32, 628–636. https://doi.org/https://dx.doi.org/10.1016/j.nut.2015.11.015

Liu, S.-Y., Zeng, F.-F., Chen, Z.-W., Wang, C.-Y., Zhao, B., Li, K.-S., 2013. Vascular endothelial growth factor gene promoter polymorphisms and Alzheimer's disease risk: A meta-analysis. CNS Neurosci. Ther., CNS Drug Reviews 19, 469–476. https://doi.org/http://dx.doi.org/10.1111/cns.12093

Liu, X., Li, L., Liu, F., Deng, S., Zhu, R., Li, Q., He, Z., 2012. ApoE gene polymorphism and vascular dementia in Chinese population: a meta-analysis. J. Neural Transm. 119, 387–394. https://doi.org/10.1007/s00702-011-0714-6

Liu, Y., Chen, Q., Liu, X., Dou, M., Li, S., Zhou, J., Liu, H., Wu, Y., Huang, Z., 2016. Genetic Association of CHAT rs3810950 and rs2177369 Polymorphisms with the Risk of Alzheimer's Disease: A Meta-Analysis. Biomed Res. Int. 2016, 1–12. https://doi.org/10.1155/2016/9418163

Lo, J.C., Groeger, J.A., Cheng, G.H., Dijk, D.J., Chee, M.W.L., 2016. Self-reported sleep duration and cognitive performance in older adults: A systematic review and meta-analysis. Sleep Med. https://doi.org/10.1016/j.sleep.2015.08.021

Loef, M., von Stillfried, N., Walach, H., 2012. Zinc diet and Alzheimer's disease: A systematic review. Nutr. Neurosci. 15, 2–12. https://doi.org/http://dx.doi.org/10.1179/1476830512Y.0000000010

Lenart-Bugla et al., 2022: What do we know about social and non-social factors influencing the pathway from cognitive health to dementia? A systematic review of reviews.

Loef, M, Walach, H., 2013. Midlife obesity and dementia: meta-analysis and adjusted forecast of dementia prevalence in the United States and China. Obesity (Silver Spring). 21, E51-5. https://doi.org/10.1002/oby.20037

Loef, Martin, Walach, H., 2013. The omega-6/omega-3 ratio and dementia or cognitive decline: a systematic review on human studies and biological evidence. J. Nutr. Gerontol. Geriatr. 32, 1–23. https://doi.org/https://dx.doi.org/10.1080/21551197.2012.752335

Loef, Martin, Walach, H., 2012. Copper and iron in Alzheimer's disease: a systematic review and its dietary implications. Br. J. Nutr. 107, 7–19. https://doi.org/https://dx.doi.org/10.1017/S000711451100376X

Loef, M, Walach, H., 2012. Fruit, vegetables and prevention of cognitive decline or dementia: a systematic review of cohort studies. J. Nutr. Health Aging 16, 626–630.

Low, L.-F., Harrison, F., Lackersteen, S.M., 2013. Does personality affect risk for dementia? A systematic review and meta-analysis. Am. J. Geriatr. Psychiatry 21, 713–728. https://doi.org/https://dx.doi.org/10.1016/j.jagp.2012.08.004

Lu, F.-P., Lin, K.-P., Kuo, H.-K., 2009. Diabetes and the risk of multi-system aging phenotypes: a systematic review and meta-analysis. PLoS One 4, e4144. https://doi.org/https://dx.doi.org/10.1371/journal.pone.0004144

Lu, Y., Liu, W., Tan, K., Peng, J., Zhu, Y., Wang, X., 2016. Genetic association of CALHM1 rs2986017 polymorphism with risk of Alzheimer's disease: A meta-analysis. Neurol. Sci., Italian Journal of Neurological Sciences 37, 525–532. https://doi.org/http://dx.doi.org/10.1007/s10072-015-2451-3

Lu, Y., Qin, X., Li, S., Zhang, X., He, Y., Peng, Q., Deng, Y., Wang, J., Xie, L., Li, T., Zeng, Z., 2014. Quantitative assessment of CYP2D6 polymorphisms and risk of Alzheimer's disease: a meta-analysis. J. Neurol. Sci. 343, 15–22. https://doi.org/https://dx.doi.org/10.1016/j.jns.2014.05.033

Lucchetta, R.C., da Mata, B.P.M., Mastroianni, P. de C., 2018. Association between Development of Dementia and Use of Benzodiazepines: A Systematic Review and Meta-analysis. Pharmacotherapy 38, 1010–1020. https://doi.org/10.1002/phar.2170

Luck, T., Luppa, M., Briel, S., Riedel-Heller, S.G., 2010. Incidence of mild cognitive impairment: A systematic review. Dement. Geriatr. Cogn. Disord., Dementia 29, 164–175. https://doi.org/http://dx.doi.org/10.1159/000272424

Lv, W., Du, N., Liu, Y., Fan, X., Wang, Y., Jia, X., Hou, X., Wang, B., 2016. Low Testosterone Level and Risk of Alzheimer's Disease in the Elderly Men: a Systematic Review and Meta-Analysis. Mol. Neurobiol. 53, 2679–2684. https://doi.org/https://dx.doi.org/10.1007/s12035-015-9315-y

Ma, L.-L., Yu, J.-T., Wang, H.-F., Meng, X.-F., Tan, C.-C., Wang, C., Tan, L., 2014. Association between cancer and Alzheimer's disease: systematic review and meta-analysis. J. Alzheimers. Dis. 42, 565–573. https://doi.org/https://dx.doi.org/10.3233/JAD-140168

Ma, Q.-P., Huang, C., Cui, Q.-Y., Yang, D.-J., Sun, K., Chen, X., Li, X.-H., 2016. Meta-Analysis of the Association between Tea Intake and the Risk of Cognitive Disorders. PLoS One 11, e0165861. https://doi.org/https://dx.doi.org/10.1371/journal.pone.0165861

MacLullich, A.M.J., Beaglehole, A., Hall, R.J., J., M.A.M., 2009. Delirium and long-term cognitive impairment. Int. Rev. Psychiatry 21, 30–42. https://doi.org/http://dx.doi.org/10.1080/09540260802675031

Makin, S.D.J., Turpin, S., Dennis, M.S., James, W.S.D., 2013. Cognitive impairment after lacunar stroke: Systematic review and meta-analysis of incidence, prevalence and comparison with other stroke subtypes. J. Neurol. Neurosurg. Psychiatry, Journal of Neurology & Psychiatry 84, 893–900. https://doi.org/http://dx.doi.org/10.1136/jnnp-2012-303645

Manley, G., Gardner, A.J., Schneider, K.J., Guskiewicz, K.M., Bailes, J., Cantu, R.C., Castellani, R.J., Turner, M., Jordan, B.D., Randolph, C., Dvorak, J., Hayden, K.A., Tator, C.H., McCrory, P., Iverson, G.L., 2017. A systematic review of potential long-term effects of sport-related concussion. Br. J. Sports Med. 51, 969–977. https://doi.org/https://dx.doi.org/10.1136/bjsports-2017-097791

Mattishent, K., Loke, Y.K., 2016. Bi-directional interaction between hypoglycaemia and cognitive impairment in elderly patients treated with glucose-lowering agents: a systematic review and meta-analysis. Diabetes. Obes. Metab. 18, 135–141. https://doi.org/https://dx.doi.org/10.1111/dom.12587

McCulloch, S., Robertson, D., Kirkpatrick, P., 2016. Sustaining people with dementia or mild cognitive impairment in employment: A systematic review of qualitative evidence. Br. J. Occup. Ther. 79, 682–692. https://doi.org/10.1177/0308022616665402

McGrattan, A. M., Zhu, Y., Richardson, C. D., Mohan, D., Soh, Y. C., Sajjad, A., ... & Stephan, B., 2021. Prevalence and risk of mild cognitive impairment in low and middle-income countries: a systematic review. Journal of Alzheimer's Disease, 79(2), 743-762.

McGuinness, B., Todd, S., Passmore, P., Bullock, R., 2009. Blood pressure lowering in patients without prior cerebrovascular disease for prevention of cognitive impairment and dementia. Cochrane Database Syst. Rev. CD004034. https://doi.org/10.1002/14651858.CD004034.pub3

Meade, T., Manolios, N., Cumming, S.R., Conaghan, P.G., Katz, P., 2018. Cognitive Impairment in Rheumatoid Arthritis: A Systematic Review. Arthritis Care Res. (Hoboken). 70, 39–52. https://doi.org/https://dx.doi.org/10.1002/acr.23243

Mendonca, M.D., Alves, L., Bugalho, M., 2016. From Subjective Cognitive Complaints to Dementia: Who Is at Risk?: A Systematic Review. Am. J. Alzheimers. Dis. Other Demen., American Journal of Alzheimer's Disease 31, 105–114. https://doi.org/10.1177/1533317515592331

Meng, X.-F., Yu, J.-T., Wang, H.-F., Tan, M.-S., Wang, C., Tan, C.-C., Tan, L., 2014. Midlife vascular risk factors and the risk of Alzheimer's disease: A systematic review and meta-analysis. J. Alzheimer's Dis. 42, 1295–1310.

Meng, X., D'Arcy, C., 2012. Education and dementia in the context of the cognitive reserve hypothesis: A systematic review with meta-analyses and qualitative analyses. PLoS One 7. https://doi.org/http://dx.doi.org/10.1371/journal.pone.0038268

Moran, G.M., Fletcher, B., Feltham, M.G., Calvert, M., Sackley, C., Marshall, T., GM, M., Fletcher, B., MG, F., Calvert, M., Sackley, C., Marshall, T., Moran, G.M., Fletcher, B., Feltham, M.G., Calvert, M., Sackley, C., Marshall, T., 2014. Fatigue, psychological and cognitive impairment following transient ischaemic attack and minor stroke: A systematic review. Eur. J. Neurol. 21, 1258–1267. https://doi.org/http://dx.doi.org/10.1111/ene.12469

Moroni, F., Ammirati, E., Magnoni, M., D'Ascenzo, F., Anselmino, M., Anzalone, N., Rocca, M.A., Falini, A., Filippi, M., Camici, P.G., 2016. Carotid atherosclerosis, silent ischemic brain damage and brain atrophy: A systematic review and meta-analysis. Int. J. Cardiol. 223, 681–687. https://doi.org/https://dx.doi.org/10.1016/j.ijcard.2016.08.234

Morra, L., Zade, D., McGlinchey, R.E., P., M.W., 2013. Normal aging and cognition: The unacknowledged contribution of cerebrovascular risk factors. Aging, Neuropsychol. Cogn., Aging & Cognition 20, 271–297. https://doi.org/http://dx.doi.org/10.1080/13825585.2012.693905

Mourao, R.J., Mansur, G., Malloy-Diniz, L.F., Castro Costa, E., Diniz, B.S., 2016. Depressive symptoms increase the risk of progression to dementia in subjects with mild cognitive impairment: systematic review and meta-analysis. Int. J. Geriatr. Psychiatry, [Comment in: Int J Geriatr Psychiatry. 2016 Nov;31(11):1255-1257; PMID: 27059938 [https://www.ncbi.nlm.nih.gov/pubmed/27059938]] 31, 905–911. https://doi.org/https://dx.doi.org/10.1002/gps.4406

Mukadam, N., Sommerlad, A., Livingston, G., 2017. The Relationship of Bilingualism Compared to Monolingualism to the Risk of Cognitive Decline or Dementia: A Systematic Review and Meta-Analysis. J. Alzheimers. Dis., [Comment in: Front Aging Neurosci. 2017 Oct 25;9:344; PMID: 29118711 [https://www.ncbi.nlm.nih.gov/pubmed/29118711]][Comment in: J Alzheimers Dis. 2017;60(4):1237-1239; PMID: 28922163 [https://www.ncbi.nlm.nih.gov/pubmed/28922163]] 58, 45–54. https://doi.org/https://dx.doi.org/10.3233/JAD-170131

Munoz Fernandez, S.S., Ivanauskas, T., Lima Ribeiro, S.M., SS, M.F., Ivanauskas, T., SM, L.R., Muñoz Fernández, S.S., Ivanauskas, T., Lima Ribeiro, S.M., 2017. Nutritional Strategies in the Management of Alzheimer Disease: Systematic Review With Network Meta-Analysis. J. Am. Med. Dir. Assoc. 18, 897.e13-897.e30. https://doi.org/10.1016/j.jamda.2017.06.015

Nader, D.A., Sanchez, Z.M., 2018. Effects of regular cannabis use on neurocognition, brain structure, and function: A systematic review of findings in adults. Am. J. Drug Alcohol Abuse 44, 4–18. https://doi.org/http://dx.doi.org/10.1080/00952990.2017.1306746

Naismith, S.L., Mowszowski, S.L., 2018. Sleep disturbance in mild cognitive impairment: A systematic review of recent findings. Curr. Opin. Psychiatry 31, 153–159. https://doi.org/http://dx.doi.org/10.1097/YCO.0000000000000397

Niu, H., Qu, Y., Li, Z., Wang, R., Li, L., Li, M., Lv, X., Gao, C., Song, Y., Li, B., 2018. Smoking and risk for Alzheimer disease: A meta-analysis based on both case-control and cohort study. J. Nerv. Ment. Dis. 206, 680–685.

Ojagbemi, A., Ffytche, D., 2016. Are stroke survivors with delirium at higher risk of post-stroke dementia? Current evidence and future directions. Int. J. Geriatr. Psychiatry 31, 1289–1294. https://doi.org/10.1002/gps.4506

Oldham, M.A., Vachon, J., Yuh, D., Lee, H.B., 2018. Cognitive outcomes after heart valve surgery: A systematic review and meta-analysis. J. Am. Geriatr. Soc. 66, 2327–2334. https://doi.org/http://dx.doi.org/10.1111/jgs.15601

Oliveira, D., Bosco, A., C, di L., 2019. Is poor health literacy a risk factor for dementia in older adults? Systematic literature review of prospective cohort studies. Maturitas 124, 8–14. https://doi.org/10.1016/j.maturitas.2019.03.010

Pal, K., Mukadam, N., Petersen, I., Cooper Kingshuk; ORCID: http://orcid.org/0000-0001-6630-6684, C.A.I.-O. http://orcid. org/Pa., 2018. Mild cognitive impairment and progression to dementia in people with diabetes, prediabetes and metabolic syndrome: A systematic review and meta-analysis. Soc. Psychiatry Psychiatr. Epidemiol. Int. J. Res. Soc. Genet. Epidemiol. Ment. Heal. Serv., Social Psychiatry 53, 1149–1160. https://doi.org/http://dx.doi.org/10.1007/s00127-018-1581-3

Pan, W., Kastin, A.J., 2014. Can sleep apnea cause Alzheimer's disease? Neurosci. Biobehav. Rev. 47, 656–669. https://doi.org/http://dx.doi.org/10.1016/j.neubiorev.2014.10.019

Lenart-Bugla et al., 2022: What do we know about social and non-social factors influencing the pathway from cognitive health to dementia? A systematic review of reviews.

Pase, M., Herbert, A., Grima, N., Pipingas, A., O'Rourke, M., 2012. Arterial stiffness as a cause of cognitive decline and dementia: a systematic review and meta-analysis. Intern. Med. J. 42, 808–815. https://doi.org/https://dx.doi.org/10.1111/j.1445-5994.2011.02645.x

Pedditizi, E., Peters, R., Beckett, N., Pedditzi, E., Peters, R., Beckett, N., 2016. The risk of overweight/obesity in mid-life and late life for the development of dementia: a systematic review and meta-analysis of longitudinal studies. Age Ageing, [Erratum in: Age Ageing. 2016 Sep;45(5):740; PMID: 27225015 [https://www.ncbi.nlm.nih.gov/pubmed/27225015]] 45, 14–21. https://doi.org/https://dx.doi.org/10.1093/ageing/afv151

Pendlebury, S.T., Rothwell, P.M., 2009. Prevalence, incidence, and factors associated with pre-stroke and post-stroke dementia: a systematic review and meta-analysis. Lancet. Neurol., [Comment in: Lancet Neurol. 2009 Nov;8(11):973-5; PMID: 19782002 [https://www.ncbi.nlm.nih.gov/pubmed/19782002]] 8, 1006–1018. https://doi.org/https://dx.doi.org/10.1016/S1474-4422(09)70236-4

Penninkilampi, R., Casey, A.N., Singh, M.F., Brodaty, H., 2018. The Association between Social Engagement, Loneliness, and Risk of Dementia: A Systematic Review and Meta-Analysis. J. Alzheimer's Dis. 66, 1619–1633. https://doi.org/10.3233/JAD-180439

Perez, L., Heim, L., Sherzai, A., Jaceldo-Siegl, K., Sherzai, A., 2012. Nutrition and vascular dementia. J. Nutr. Health Aging 16, 319–324.

Peters, R., Peters, J., Booth, A., Mudway, I., 2015. Is air pollution associated with increased risk of cognitive decline? A systematic review. Age Ageing 44, 755–760. https://doi.org/https://dx.doi.org/10.1093/ageing/afv087

Petersson, S.D., Philippou, E., 2016. Mediterranean Diet, Cognitive Function, and Dementia: A Systematic Review of the Evidence. Adv. Nutr. 7, 889–904. https://doi.org/https://dx.doi.org/10.3945/an.116.012138

Piazza-Gardner, A.K., Gaffud, T.J.B., Barry, A.E., 2013. The impact of alcohol on Alzheimer's disease: a systematic review. Aging Ment. Health 17, 133–146. https://doi.org/https://dx.doi.org/10.1080/13607863.2012.742488

Plassman, B.L., Williams, J.W.J., Burke, J.R., Holsinger, T., Benjamin, S., 2010. Systematic review: factors associated with risk for and possible prevention of cognitive decline in later life. Ann. Intern. Med. 153, 182–193. https://doi.org/https://dx.doi.org/10.7326/0003-4819-153-3-201008030-00258

Power, M.C., Weuve, J., Gagne, J.J., McQueen, M.B., Viswanathan, A., Blacker, D., 2011. The association between blood pressure and incident Alzheimer disease: a systematic review and meta-analysis. Epidemiology, [Comment in: Epidemiology. 2012 Jan;23(1):176-7; author reply 177-8; PMID: 22157315 [https://www.ncbi.nlm.nih.gov/pubmed/22157315]] 22, 646–659. https://doi.org/https://dx.doi.org/10.1097/EDE.0b013e31822708b5

Proietti, R., Manzoni, G.M., Cravello, L., Castelnuovo, G., Bernier, M.L., Essebag, V., 2014. Can cardiac resynchronization therapy improve cognitive function? A systematic review. Pacing Clin. Electrophysiol. 37, 520–530. https://doi.org/https://dx.doi.org/10.1111/pace.12328

Purnell, C., Gao, S., Callahan, C.M., Hendrie, H.C., 2009. Cardiovascular risk factors and incident Alzheimer disease: A systematic review of the literature. Alzheimer Dis. Assoc. Disord. 23, 1–10. https://doi.org/http://dx.doi.org/10.1097/WAD.0b013e318187541c

Qin, X., Peng, Q., Zeng, Z., Chen, Z., Lin, L., Deng, Y., Huang, X., Xu, J., Wu, H., Huang, S., Li, S., Zhao, J., 2012. Interleukin-1A -889C/T polymorphism and risk of Alzheimer's disease: a meta-analysis

based on 32 case-control studies. J. Neurol. 259, 1519–1529. https://doi.org/https://dx.doi.org/10.1007/s00415-011-6381-6

Quinn, T.J., Gallacher, J., Deary, I.J., Lowe, G.D.O., Fenton, C., Stott, D.J., 2011. Association between circulating hemostatic measures and dementia or cognitive impairment: systematic review and meta-analyzes. J. Thromb. Haemost. 9, 1475–1482. https://doi.org/https://dx.doi.org/10.1111/j.1538-7836.2011.04403.x

Rai, V., 2017. Methylenetetrahydrofolate Reductase (MTHFR) C677T Polymorphism and Alzheimer Disease Risk: a Meta-Analysis. Mol. Neurobiol. 54, 1173–1186. https://doi.org/https://dx.doi.org/10.1007/s12035-016-9722-8

Rayes, H. Al, Tani, C., Kwan, A., Marzouk, S., Colosimo, K., Medina-Rosas, J., Mustafa, A., Su, J., Lambiris, P., Mosca, M., Touma, Z., 2018. What is the prevalence of cognitive impairment in lupus and which instruments are used to measure it? A systematic review and meta-analysis. Semin. Arthritis Rheum. 48, 240–255. https://doi.org/https://dx.doi.org/10.1016/j.semarthrit.2018.02.007

Rensma, S.P., van Sloten, T.T., Launer, L.J., Stehouwer, C.D.A., 2018. Cerebral small vessel disease and risk of incident stroke, dementia and depression, and all-cause mortality: A systematic review and meta-analysis. Neurosci. Biobehav. Rev. 90, 164–173. https://doi.org/https://dx.doi.org/10.1016/j.neubiorev.2018.04.003

Ryman, D.C., Acosta-Baena, N., Aisen, P.S., Bird, T., Danek, A., Fox, N.C., Goate, A., Frommelt, P., Ghetti, B., Langbaum, J.B.S., Lopera, F., Martins, R., Masters, C.L., Mayeux, R.P., McDade, E., Moreno, S., Reiman, E.M., Ringman, J.M., Salloway, S., Schofield, P.R., Sperling, R., Tariot, P.N., Xiong, C., Morris, J.C., C., B.N., 2014. Symptom onset in autosomal dominant Alzheimer disease: A systematic review and meta-analysis. Neurology 83, 253–260. https://doi.org/http://dx.doi.org/10.1212/WNL.0000000000000596

Sajeev, G., Weuve, J., Jackson, J.W., VanderWeele, T.J., Bennett, D.A., Grodstein, F., Blacker, D., 2016. Late-life Cognitive Activity and Dementia: A Systematic Review and Bias Analysis. Epidemiology 27, 732–742. https://doi.org/https://dx.doi.org/10.1097/EDE.0000000000000513

Sakusic, A., JC, O., Dziadzko, M., Volha, D., Ali, R., TD, S., Kashyap, R., AM, F., JD, F., Petersen, R., Gajic, O., AA, R., 2018. Potentially Modifiable Risk Factors for Long-Term Cognitive Impairment After Critical Illness: A Systematic Review. Mayo Clin. Proc. 93, 68–82. https://doi.org/10.1016/j.mayocp.2017.11.005

Santabárbara, J., DM, L., Villagrasa, B., Lobo, E., Lopez-Anton, R., Santabarbara, J., Lipnicki, D.M., Villagrasa, B., Lobo, E., Lopez-Anton, R., Santabárbara, J., DM, L., Villagrasa, B., Lobo, E., Lopez-Anton, R., 2019. Anxiety and risk of dementia: Systematic review and meta-analysis of prospective cohort studies. Maturitas 119, 14–20. https://doi.org/https://dx.doi.org/10.1016/j.maturitas.2018.10.014

Savva, G.M., Stephan, B.C.M., Group, A.S.V.D.S.R., 2010. Epidemiological studies of the effect of stroke on incident dementia: a systematic review. Stroke 41, e41-6. https://doi.org/https://dx.doi.org/10.1161/STROKEAHA.109.559880

Seifan, A., Schelke, M., Obeng-Aduasare, Y., Alon, I., 2015. Early life epidemiology of Alzheimer’s Disease-A critical review. Neuroepidemiology 45, 237–254. https://doi.org/http://dx.doi.org/10.1159/000439568

Seitz, D.P., Shah, P.S., Herrmann, N., Beyene, J., Siddiqui, N., 2011. Exposure to general anesthesia and risk of Alzheimer’s disease: a systematic review and meta-analysis. BMC Geriatr. 11, 83. https://doi.org/https://dx.doi.org/10.1186/1471-2318-11-83

Lenart-Bugla et al., 2022: What do we know about social and non-social factors influencing the pathway from cognitive health to dementia? A systematic review of reviews.

Sekhon, H., Allali, G., Launay, C.P., Barden, J., Szturm, T., Liu-Ambrose, T., Chester, V.L., Wong, C.H., Beauchet, O., Consortium, C.G., 2019. Motoric cognitive risk syndrome, incident cognitive impairment and morphological brain abnormalities: Systematic review and meta-analysis. Maturitas 123, 45–54. https://doi.org/https://dx.doi.org/10.1016/j.maturitas.2019.02.006

Sharp, E.S., Gatz, M., 2011. Relationship between education and dementia: an updated systematic review. Alzheimer Dis. Assoc. Disord. 25, 289–304. https://doi.org/https://dx.doi.org/10.1097/WAD.0b013e318211c83c

Sharp, S.I., Aarsland, D., Day, S., Sonnesyn, H., Ballard, C., 2011. Hypertension is a potential risk factor for vascular dementia: Systematic review. Int. J. Geriatr. Psychiatry 26, 661–669. https://doi.org/10.1002/gps.2572

Shen, L., Ji, H.-F., 2015. Vitamin D deficiency is associated with increased risk of Alzheimer's disease and dementia: evidence from meta-analysis. Nutr. J. 14, 76. https://doi.org/https://dx.doi.org/10.1186/s12937-015-0063-7

Shen, Z., Ruan, Q., Yu, Z., Sun, Z., 2017. Chronic kidney disease-related physical frailty and cognitive impairment: A systemic review. Geriatr. Gerontol. Int. 17, 529–544. https://doi.org/http://dx.doi.org/10.1111/ggi.12758

Shi, L., Chen, S.-J., Ma, M.-Y., Bao, Y.-P., Han, Y., Wang, Y.-M., Shi, J., Vitiello, M. V, Lu, L., 2018. Sleep disturbances increase the risk of dementia: A systematic review and meta-analysis. Sleep Med. Rev., [Comment in: Sleep Med Rev. 2019 Feb;43:22; PMID: 30503714 [https://www.ncbi.nlm.nih.gov/pubmed/30503714]][Comment in: Sleep Med Rev. 2019 Feb;43:133-134; PMID: 30473232 [https://www.ncbi.nlm.nih.gov/pubmed/30473232]] 40, 4–16. https://doi.org/https://dx.doi.org/10.1016/j.smrv.2017.06.010

Shi, L., Zhao, L., Wong, A., Wang, D., Mok, V., 2015. Mapping the Relationship of Contributing Factors for Preclinical Alzheimer's Disease. Sci. Rep. 5, 11259. https://doi.org/https://dx.doi.org/10.1038/srep11259

Siervo, M., Arnold, R., Wells, J.C.K., Tagliabue, A., Colantuoni, A., Albanese, E., Brayne, C., Stephan, B.C.M., 2011. Intentional weight loss in overweight and obese individuals and cognitive function: a systematic review and meta-analysis. Obes. Rev. 12, 968–983. https://doi.org/https://dx.doi.org/10.1111/j.1467-789X.2011.00903.x

Snowden, M., Steinman, L., Mochan, K., Grodstein, F., Prohaska, T.R., Thurman, D.J., Brown, D.R., Laditka, J.N., Soares, J., Zweiback, D.J., Little, D., Anderson, L.A., 2011. Effect of exercise on cognitive performance in community-dwelling older adults: Review of intervention trials and recommendations for public health practice and research. J. Am. Geriatr. Soc. 59, 704–716. https://doi.org/http://dx.doi.org/10.1111/j.1532-5415.2011.03323.x

Solfrizzi, V., Custodero, C., Lozupone, M., Imbimbo, B.P., Valiani, V., Agosti, P., Schilardi, A., D'Introno, A., La Montagna, M., Calvani, M., Guerra, V., Sardone, R., Abbrescia, D.I., Bellomo, A., Greco, A., Daniele, A., Seripa, D., Logroscino, G., Sabba, C., Panza, F., 2017. Relationships of Dietary Patterns, Foods, and Micro- and Macronutrients with Alzheimer's Disease and Late-Life Cognitive Disorders: A Systematic Review. J. Alzheimers. Dis. 59, 815–849. https://doi.org/https://dx.doi.org/10.3233/JAD-170248

Sommer, I., Griebler, U., Kien, C., Auer, S., Klerings, I., Hammer, R., Holzer, P., Gartlehner, G., 2017. Vitamin D deficiency as a risk factor for dementia: a systematic review and meta-analysis. BMC Geriatr. 17, 16. https://doi.org/https://dx.doi.org/10.1186/s12877-016-0405-0

Sommerlad, A., Ruegger, J., Singh-Manoux, A., Lewis, G., Livingston, G., 2017. Marriage and risk of dementia: Systematic review and meta-analysis of observational studies. J. Neurol. Neurosurg. Psychiatry, Journal of Neurology & Psychiatry 89, 231–238.

Song, Y.-N., Wang, P., Xu, W., Li, J.-Q., Cao, X.-P., Yu, J.-T., Tan, L., 2018. Risk factors of rapid cognitive decline in Alzheimer's disease and mild cognitive impairment: A systematic review and meta-analysis. J. Alzheimer's Dis. 66, 497–515. https://doi.org/http://dx.doi.org/10.3233/JAD-180476

Spano, M., Signorelli, M., Vitaliani, R., Aguglia, E., Glometto, B., 2015. The possible involvement of mitochondrial dysfunctions in Lewy body dementia: A systematic review. Funct. Neurol. 30, 151–158.

Stacey, D., Ciobanu, L.G., Baune, B.T., 2017. A systematic review on the association between inflammatory genes and cognitive decline in non-demented elderly individuals. Eur. Neuropsychopharmacol. 27, 568–588. https://doi.org/http://dx.doi.org/10.1016/j.euroneuro.2015.12.017

Stefanidis, K.B., Askew, C.D., Greaves, K., Summers, M.J., 2018. The Effect of Non-Stroke Cardiovascular Disease States on Risk for Cognitive Decline and Dementia: A Systematic and Meta-Analytic Review. Neuropsychol. Rev. 28, 1–15. https://doi.org/https://dx.doi.org/10.1007/s11065-017-9359-z

Stephen, R., Hongisto, K., Solomon, A., Lonnroos, E., 2017. Physical activity and Alzheimer's disease: A systematic review. Journals Gerontol. Ser. A Biol. Sci. Med. Sci., Journal of Gerontology 72, 733–739.

Stewart, M.W., Traylor, A.C., Bratzke, L.C., 2015. Nutrition and Cognition in Older Adults With Heart Failure: A Systematic Review. J. Gerontol. Nurs. 41, 50–59. https://doi.org/https://dx.doi.org/10.3928/00989134-20151015-06

Stirland, L.E., O'Shea, C.I., Russ, T.C., 2018. Passive smoking as a risk factor for dementia and cognitive impairment: systematic review of observational studies. Int. psychogeriatrics 30, 1177–1187. https://doi.org/https://dx.doi.org/10.1017/S1041610217002824

Stocker, H., Mollers, T., Perna, L., Brenner, H., 2018. The genetic risk of Alzheimer's disease beyond APOE epsilon4: systematic review of Alzheimer's genetic risk scores. Transl. Psychiatry 8, 166. https://doi.org/https://dx.doi.org/10.1038/s41398-018-0221-8

Su, W.-H., Shi, Z.-H., Liu, S.-L., Wang, X.-D., Liu, S., Ji, Y., 2017. Updated meta-analysis of the role of APOE epsilon2/epsilon3/epsilon4 alleles in frontotemporal lobar degeneration. Oncotarget 8, 43721–43732. https://doi.org/https://dx.doi.org/10.18632/oncotarget.17341

Subota, A., Pham, T., Jette, N., Sauro, K., Lorenzetti, D., Holroyd-Leduc, J., 2017. The association between dementia and epilepsy: A systematic review and meta-analysis. Epilepsia 58, 962–972. https://doi.org/https://dx.doi.org/10.1111/epi.13744

Sun, J.-H., Tan, Lan, Wang, H.-F., Tan, M.-S., Tan, Lin, Li, J.-Q., Xu, W., Zhu, X.-C., Jiang, T., Yu, J.-T., 2015. Genetics of Vascular Dementia: Systematic Review and Meta-Analysis. J. Alzheimers. Dis. 46, 611–629. https://doi.org/https://dx.doi.org/10.3233/JAD-143102

Sun, R., Yang, S., Zheng, B., Liu, J., Ma, X., 2019. Apolipoprotein E Polymorphisms and Parkinson Disease With or Without Dementia: A Meta-Analysis Including 6453 Participants. J. Geriatr. Psychiatry Neurol. 32, 891988718813675. https://doi.org/10.1177/0891988718813675

Tada, A., Miura, H., 2017. Association between mastication and cognitive status: A systematic review. Arch. Gerontol. Geriatr. 70, 44–53. https://doi.org/http://dx.doi.org/10.1016/j.archger.2016.12.006

Tan, E., Köhler, S., Hamel, R., Muñoz-Sánchez, J., Verhey, F., Ramakers, I., 2019. Depressive Symptoms in Mild Cognitive Impairment and the Risk of Dementia: A Systematic Review and Comparative Meta-Analysis of Clinical and Community-Based Studies. J. Alzheimers. Dis. 67, 1319–1329. https://doi.org/10.3233/JAD-180513

Tonsekar, P.P., Jiang, S.S., Yue, G., 2017. Periodontal disease, tooth loss and dementia: Is there a link? A systematic review. Gerodontology 34, 151–163. https://doi.org/https://dx.doi.org/10.1111/ger.12261

Udompanich, S., GY, L., Apostolakis, S., DA, L., 2013. Atrial fibrillation as a risk factor for cognitive impairment: a semi-systematic review. QJM 106, 795–802. https://doi.org/10.1093/qjmed/hct129

Ungprasert, P., Wijarnpreecha, K., Thongprayoon, C., 2016. Rheumatoid arthritis and the risk of dementia: A systematic review and meta-analysis. Neurol. India 64, 56–61. https://doi.org/https://dx.doi.org/10.4103/0028-3886.173623

Vagelatos, N.T., Eslick, G.D., NT, V., GD, E., Vagelatos, N.T., Eslick, G.D., NT, V., GD, E., Vagelatos, N.T., Eslick, G.D., 2013. Type 2 diabetes as a risk factor for Alzheimer's disease: the confounders, interactions, and neuropathology associated with this relationship. Epidemiol. Rev. 35, 152–160. https://doi.org/10.1093/epirev/mxs012

van Dalen, J.W., van Wanrooij, L.L., van Charante, E.P.M., Brayne, C., van Gool, W.A., Richard Jan Willem; ORCID: http://orcid.org/0000-0002-3439-8841, E.A.I.-O. http://orcid. org/va. D., 2018. Association of apathy with risk of incident dementia: A systematic review and meta-analysis. JAMA Psychiatry, A.M.A. Archives of General Psychiatry, Archives of General Psychiatry 75, 1012–1021. https://doi.org/http://dx.doi.org/10.1001/jamapsychiatry.2018.1877

Van Dam, F., Van Gool, W.A., 2009. Hyperhomocysteinemia and Alzheimer's disease: A systematic review. Arch. Gerontol. Geriatr. 48, 425–430. https://doi.org/http://dx.doi.org/10.1016/j.archger.2008.03.009

van de Rest, O., Berendsen, A.A., Haveman-Nies, A., de Groot, L.C., 2015. Dietary patterns, cognitive decline, and dementia: a systematic review. Adv. Nutr. 6, 154–168. https://doi.org/https://dx.doi.org/10.3945/an.114.007617

van den Berg, E., Kloppenborg, R.P., Kessels, R.P.C., Kappelle, L.J., Biessels, G.J., 2009. Type 2 diabetes mellitus, hypertension, dyslipidemia and obesity: A systematic comparison of their impact on cognition. Biochim. Biophys. Acta 1792, 470–481. https://doi.org/https://dx.doi.org/10.1016/j.bbadis.2008.09.004

van der Velpen, I.F., Feleus, S., Bertens, A.S., Sabayan Behnam; ORCID: http://orcid.org/0000-0002-1176-9152, B.A.I.-O. http://orcid. org/Sabaya., 2017. Hemodynamic and serum cardiac markers and risk of cognitive impairment and dementia. Alzheimer's Dement. J. Alzheimer's Assoc. 13, 441–453. https://doi.org/http://dx.doi.org/10.1016/j.jalz.2016.09.004

van Rijsbergen, M.W.A., Mark, R.E., de Kort, P.L.M., Sitskoorn, M.M., 2014. Subjective cognitive complaints after stroke: a systematic review. J. Stroke Cerebrovasc. Dis. 23, 408–420. https://doi.org/https://dx.doi.org/10.1016/j.jstrokecerebrovasdis.2013.05.003

van Rooij, F.G., Kessels, R.P.C., Richard, E., De Leeuw, F.-E., van Dijk, E.J., 2016. Cognitive Impairment in Transient Ischemic Attack Patients: A Systematic Review. Cerebrovasc. Dis. 42, 1–9. https://doi.org/https://dx.doi.org/10.1159/000444282

van Sloten, T.T., Protogerou, A.D., Henry, R.M.A., Schram, M.T., Launer, L.J., Stehouwer Miranda T.; ORCID: http://orcid.org/0000-0003-0515-4124, C.D.A.A.I.-O. http://orcid. org/Schra., 2015. Association between arterial stiffness, cerebral small vessel disease and cognitive impairment: A

systematic review and meta-analysis. Neurosci. Biobehav. Rev. 53, 121–130. https://doi.org/http://dx.doi.org/10.1016/j.neubiorev.2015.03.011

Veronese, N., Zurlo, A., Solmi, M., Luchini, C., Trevisan, C., Bano, G., Manzato, E., Sergi, G., Rylander, R., 2016. Magnesium status in Alzheimer's disease: A systematic review. Am. J. Alzheimers. Dis. Other Demen., American Journal of Alzheimer's Disease 31, 208–213. https://doi.org/http://dx.doi.org/10.1177/1533317515602674

Vnukova, M., Ptacek, R., Raboch, J., Stefano, G.B., 2017. Decreased Central Nervous System Grey Matter Volume (GMV) in Smokers Affects Cognitive Abilities: A Systematic Review. Med. Sci. Monit. 23, 1907–1915.

Walker, J. D., Spiro, G., Loewen, K., & Jacklin, K., 2020. Alzheimer's disease and related dementia in Indigenous populations: a systematic review of risk factors. Journal of Alzheimer's Disease, 78(4), 1439-1451.

Wang, C., Yu, J.-T., Wang, H.-F., Jiang, T., Tan, C.-C., Meng, X.-F., Soares, H.D., Tan, L., 2014. Meta-analysis of peripheral blood apolipoprotein E levels in Alzheimer's disease. PLoS One 9, e89041. https://doi.org/https://dx.doi.org/10.1371/journal.pone.0089041

Wang, H.X., Xu, W., Pei, J.J., 2012. Leisure activities, cognition and dementia. Biochim. Biophys. Acta - Mol. Basis Dis. 1822, 482–491. https://doi.org/10.1016/j.bbadis.2011.09.002

Wang, J., Xu, W., Sun, S., Yu, S., Fan, L., 2018. Headache disorder and the risk of dementia: a systematic review and meta-analysis of cohort studies. J. Headache Pain 19, 95. https://doi.org/https://dx.doi.org/10.1186/s10194-018-0925-4

Wang, Q., Liu, J., Guo, Y., Dong, G., Zou, W., Chen, Z., 2019. Association between BDNF G196A (Val66Met) polymorphism and cognitive impairment in patients with Parkinson's disease: a meta-analysis. Brazilian J. Med. Biol. Res. = Rev. Bras. Pesqui. medicas e Biol. 52, e8443. https://doi.org/10.1590/1414-431X20198443

Wang, X.-J., Xu, W., Li, J.-Q., Cao, X.-P., Tan, L., Yu, J.-T., 2019. Early-life risk factors for dementia and cognitive impairment in later life: A systematic review and meta-analysis. J. Alzheimer's Dis. 67, 221–229. https://doi.org/http://dx.doi.org/10.3233/JAD-180856

Wang, X., Cui, N., Yang, J., Qiu, X., Gao, J., Yang, N., Zheng, F., 2014. Angiotensin-converting enzyme insertion/deletion polymorphism is not a major determining factor in the development of sporadic Alzheimer disease: evidence from an updated meta-analysis. PLoS One 9, e111406. https://doi.org/https://dx.doi.org/10.1371/journal.pone.0111406

Wang, X.J., Xu, W., Li, J.Q., Cao, X.P., Tan, L., Yu, J.T., 2019. Early-Life Risk Factors for Dementia and Cognitive Impairment in Later Life: A Systematic Review and Meta-Analysis. J. Alzheimer's Dis. https://doi.org/10.3233/JAD-180856

Wang, Y.-C., Tai, P.-A., Poly, T.N., Islam, M.M., Yang, H.-C., Wu, C.-C., Li, Y.-C.J., 2018. Increased Risk of Dementia in Patients with Antidepressants: A Meta-Analysis of Observational Studies. Behav. Neurol. 2018, 5315098. https://doi.org/https://dx.doi.org/10.1155/2018/5315098

Wang, Y., Liu, S., Wang, J., Zhang, J., Hua, Y., Li, H., Tan, H., Kuai, B., Wang, B., Sheng, S., 2017. Association between LRP1 C766T polymorphism and Alzheimer's disease susceptibility: a meta-analysis. Sci. Rep. 7, 8435. https://doi.org/https://dx.doi.org/10.1038/s41598-017-08335-w

Lenart-Bugla et al., 2022: What do we know about social and non-social factors influencing the pathway from cognitive health to dementia? A systematic review of reviews.

Wang, Y., Xu, S., Liu, Z., Lai, C., Xie, Z., Zhao, C., Wei, Y., Bi, J., 2013. Meta-analysis on the association between the TF gene rs1049296 and AD. Can. J. Neurol. Sci. / Le J. Can. Des Sci. Neurol. 40, 691–697. https://doi.org/http://dx.doi.org/10.1017/S0317167100014931

Wang, Yunyang, Sheng, Q., Hou, X., Wang, B., Zhao, W., Yan, S., Wang, Yangang, Zhao, S., 2016. Thyrotropin and Alzheimer's Disease Risk in the Elderly: a Systematic Review and Meta-Analysis. Mol. Neurobiol. 53, 1229–1236. https://doi.org/https://dx.doi.org/10.1007/s12035-014-9078-x

Weber, A., Mak, S.H., Berenbaum, F., Sellam, J., Zheng, Y.-P., Han, Y., Wen, C., 2019. Association between osteoarthritis and increased risk of dementia: A systemic review and meta-analysis. Medicine (Baltimore). 98, e14355. https://doi.org/https://dx.doi.org/10.1097/MD.0000000000014355

Wu, Lei, Sun, D., He, Y., 2017. Coffee intake and the incident risk of cognitive disorders: A dose-response meta-analysis of nine prospective cohort studies. Clin. Nutr. 36, 730–736. https://doi.org/https://dx.doi.org/10.1016/j.clnu.2016.05.015

Wu, L., Sun, D., Tan, Y., 2018. A systematic review and dose-response meta-analysis of sleep duration and the occurrence of cognitive disorders. Sleep Breath. 22, 805–814. https://doi.org/https://dx.doi.org/10.1007/s11325-017-1527-0

Wu, L, Sun, D., Tan, Y., 2017. Intake of Fruit and Vegetables and the Incident Risk of Cognitive Disorders: A Systematic Review and Meta-Analysis of Cohort Studies. J. Nutr. Health Aging 21, 1284–1290. https://doi.org/https://dx.doi.org/10.1007/s12603-017-0875-6

Wu, S., Ding, Y., Wu, F., Li, R., Hou, J., Mao, P., 2015. Omega-3 fatty acids intake and risks of dementia and Alzheimer's disease: A meta-analysis. Neurosci. Biobehav. Rev. 48, 1–9. https://doi.org/http://dx.doi.org/10.1016/j.neubiorev.2014.11.008

Wu, W., Jiang, H., Wang, M., Zhang, D., 2013. Meta-analysis of the association between urokinase-plasminogen activator gene rs2227564 polymorphism and Alzheimer's disease. Am. J. Alzheimers. Dis. Other Demen., American Journal of Alzheimer's Disease 28, 517–523. https://doi.org/http://dx.doi.org/10.1177/1533317513494450

Wu, Y.-T., Prina, A.M., Brayne, C., 2015. The association between community environment and cognitive function: a systematic review. Soc. Psychiatry Psychiatr. Epidemiol. 50, 351–362. https://doi.org/https://dx.doi.org/10.1007/s00127-014-0945-6

Wu, Z., Nakanishi, H., 2014. Connection between periodontitis and Alzheimer's disease: possible roles of microglia and leptomeningeal cells. J. Pharmacol. Sci. 126, 8–13.

Wu, Z., Phyo, A. Z. Z., Al-Harbi, T., Woods, R. L., & Ryan, J., 2020. Distinct cognitive trajectories in late life and associated predictors and outcomes: a systematic review. Journal of Alzheimer's disease reports, 4(1), 459-478.

X., Z., Y., Z., 2018. Sleep-disordered breathing and the risk of cognitive decline: a meta-analysis of 19,940 participants. Sleep Breath. 22, 1–9. https://doi.org/10.1007/s11325-017-1562-x

Xu, H.-M., Tan, Lin, Wan, Y., Tan, M.-S., Zhang, W., Zheng, Z.-J., Kong, L.-L., Wang, Z.-X., Jiang, T., Tan, Lan, Yu, J.-T., 2017. Pgrn is associated with late-onset alzheimer's disease: A case–control replication study and meta-analysis. Mol. Neurobiol. 54, 1–9. https://doi.org/10.1007/s12035-016-9698-4

Xu, W., Wang, H., Wan, Y., Tan, C., Li, J., Tan, L., Yu, J.-T., 2017. Alcohol consumption and dementia risk: a dose-response meta-analysis of prospective studies. Eur. J. Epidemiol., [Comment in: Eur J Epidemiol. 2017 Jul;32(7):627-629; PMID: 28508266

[https://www.ncbi.nlm.nih.gov/pubmed/28508266]] 32, 31–42. https://doi.org/https://dx.doi.org/10.1007/s10654-017-0225-3

Yan, D., Zhang, Y., Liu, L., Yan, H., 2016. Pesticide exposure and risk of Alzheimer's disease: a systematic review and meta-analysis. Sci. Rep. 6, 32222. https://doi.org/https://dx.doi.org/10.1038/srep32222

Yang, D., Liu, C., Shi, J., Wang, N., Du, X., Yin, Q., Wang, Y., 2014. Association of XRCC1 Arg399Gln polymorphism with bladder cancer susceptibility: a meta-analysis. Gene 534, 17–23. https://doi.org/10.1016/j.gene.2013.10.038

Yates, J.A., Clare, L., Woods, R.T., 2013. Mild cognitive impairment and mood: a systematic review. Rev. Clin. Gerontol. 23, 317–356. https://doi.org/10.1017/S0959259813000129

Yates, L.A., Ziser, S., Spector, A., Orrell, M., 2016. Cognitive leisure activities and future risk of cognitive impairment and dementia: systematic review and meta-analysis. Int. psychogeriatrics 28, 1791–1806.

Yuan, H., Du, L., Ge, P., Wang, X., Xia, Q., 2018. Association of microtubule-associated protein tau gene polymorphisms with the risk of sporadic Alzheimer's disease: a meta-analysis. Int. J. Neurosci. 128, 577–585. https://doi.org/https://dx.doi.org/10.1080/00207454.2017.1400972

Yuan, X.-Y., Wang, X.-G., 2017. Mild cognitive impairment in type 2 diabetes mellitus and related risk factors: A review. Rev. Neurosci. 28, 715–723. https://doi.org/http://dx.doi.org/10.1515/revneuro-2017-0016

Yuan, Y., Piao, J., Ma, K., Lu, N., 2015. Angiotensin-converting enzyme gene insertion-deletion polymorphism is a risk marker for Alzheimer's disease in a Chinese population: a meta-analysis of case-control studies. J. Neural Transm. 122, 1105–1113. https://doi.org/https://dx.doi.org/10.1007/s00702-015-1368-6

Yusufov, M., Weyandt, L.L., Piryatinsky, I., 2017. Alzheimer's disease and diet: a systematic review. Int. J. Neurosci. 127, 161–175.

Zeng, L.-F., Cao, Y., Liang, W.-X., Bao, W.-H., Pan, J.-K., Wang, Q., Liu, J., Liang, H.-D., Xie, H., Chai, Y.-T., Guan, Z.-T., Cao, Q., Li, X.-Y., Yang, L., Xu, W.-H., Mi, S.-Q., Wang, N.-S., 2017. An exploration of the role of a fish-oriented diet in cognitive decline: a systematic review of the literature. Oncotarget 8, 39877–39895. https://doi.org/https://dx.doi.org/10.18632/oncotarget.16347

Zhang, D.-M., Ye, J.-X., Mu, J.-S., Cui, X.-P., D.-M., Z., J.-X., Y., J.-S., M., X.-P., C., 2017. Efficacy of Vitamin B Supplementation on Cognition in Elderly Patients with Cognitive-Related Diseases: A Systematic Review and Meta-Analysis. J. Geriatr. Psychiatry Neurol. 30, 50–59. https://doi.org/10.1177/0891988716673466

Zhang, J., Chen, C., Hua, S., Liao, H., Wang, M., Xiong, Y., Cao, F., 2017. An updated meta-analysis of cohort studies: Diabetes and risk of Alzheimer's disease. Diabetes Res. Clin. Pract. 124, 41–47. https://doi.org/10.1016/j.diabres.2016.10.024

Zhang, X., Cai, X., Shi, X., Zheng, Z., Zhang, A., Guo, J., Fang, Y., 2016. Chronic obstructive pulmonary disease as a risk factor for cognitive dysfunction: A meta-analysis of current studies. J. Alzheimer's Dis. 52, 101–111. https://doi.org/10.3233/JAD-150735

Zhang, Y., Chen, J., Qiu, J., Li, Y., Wang, J., Jiao, J., 2016. Intakes of fish and polyunsaturated fatty acids and mild-to-severe cognitive impairment risks: a dose-response meta-analysis of 21 cohort studies. Am. J. Clin. Nutr., [Comment in: Am J Clin Nutr. 2016 Aug;104(2):537; PMID: 27481867

[https://www.ncbi.nlm.nih.gov/pubmed/27481867]][Comment in: Am J Clin Nutr. 2016 Aug;104(2):537-8; PMID: 27481868 [https://www.ncbi.nlm.nih.gov/pubmed/27481868]] 103, 330–340. https://doi.org/https://dx.doi.org/10.3945/ajcn.115.124081

Zheng, J.-J., Li, W.-X., Liu, J.-Q., Guo, Y.-C., Wang, Q., Li, G.-H., Dai, S.-X., Huang, J.-F., 2018. Low expression of aging-related NRXN3 is associated with Alzheimer disease: A systematic review and meta-analysis. Medicine (Baltimore). 97, e11343. https://doi.org/https://dx.doi.org/10.1097/MD.0000000000011343

Zheng, Y., Fan, S., Liao, W., Fang, W., Xiao, S., Liu, J., 2017. Hearing impairment and risk of Alzheimer's disease: a meta-analysis of prospective cohort studies. Neurol. Sci. 38, 233–239. https://doi.org/https://dx.doi.org/10.1007/s10072-016-2779-3

Zhong, G., Wang, Y., Zhang, Y., Guo, J.J., Zhao, Y., 2015. Smoking is associated with an increased risk of dementia: a meta-analysis of prospective cohort studies with investigation of potential effect modifiers. PLoS One, [Erratum in: PLoS One. 2015;10(4):e0126169; PMID: 25875505 [https://www.ncbi.nlm.nih.gov/pubmed/25875505]] 10, e0118333. https://doi.org/https://dx.doi.org/10.1371/journal.pone.0118333

Zhou, F., Chen, S., 2019. Hyperhomocysteinemia and risk of incident cognitive outcomes: an updated dose-response meta-analysis of prospective cohort studies. Ageing Res. Rev. 51, 55–66. https://doi.org/10.1016/j.arr.2019.02.006

Zhou, J., Yu, J.-T., Wang, H.-F., Meng, X.-F., Tan, C.-C., Wang, J., Wang, C., Tan, L., 2015. Association between stroke and Alzheimer's disease: Systematic review and meta-analysis. J. Alzheimer's Dis. 43, 479–489.

Zhou, Q., Zhao, F., Lv, Z., Zheng, C., Zheng, W., Sun, L., Wang, N., Pang, S., de Andrade, F.M., Fu, M., He, X., Hui, J., Jiang, W., Yang, C., Shi, X., Zhu, X., Pang, G., Yang, Y., Xie, H., Zhang, W., Hu, C., Yang, Z., 2014. Association between APOC1 polymorphism and Alzheimer's disease: A case-control study and meta-analysis. PLoS One 9.

Zhou, S., Tan, C., Hou, X., Cao, X., Tan, L., Yu, J., 2019. TREM2 Variants and Neurodegenerative Diseases: A Systematic Review and Meta-Analysis. J. Alzheimers. Dis. 68, 1171–1184. https://doi.org/10.3233/JAD-181038

Zhu, R., Liu, X., He, Z., 2017. The Bridging Integrator 1 Gene Polymorphism rs744373 and the Risk of Alzheimer's Disease in Caucasian and Asian Populations: An Updated Meta-Analysis. Mol. Neurobiol. 54, 1419–1428. https://doi.org/https://dx.doi.org/10.1007/s12035-016-9760-2
