# Supplementary material for: What Do We Know about Social and Non-Social Factors Influencing the Pathway from Cognitive Health to Dementia? A Systematic Review of Reviews
Source: Brain Sci. 2022 Sep 8;12(9):1214. doi: 10.3390/brainsci12091214 (PMC9497077; doi:10.3390/brainsci12091214)
Supplement: Supplementary file 1 [file brainsci-12-01214-s001.zip › Table S2. Search strategies.pdf]

Lenart-Bugla et al., 2022: What do we know about social and non-social factors influencing the pathway from cognitive health to dementia? A systematic review of reviews.

Supplementary material
Table S2: Search strategies.

Database	Command	Criteria	Results
CINAHL Complete	All: Abstract<br>Dementia* OR Alzheimer* OR (Cognitive Impairment) OR (Cognitive decline*) OR (Cognitive function*) OR (Cognitive reserve) OR (Cognitive capacit*) AND (risk factor* OR predictive factor* OR prognostic factor* OR course OR cause OR contributing factor* OR predisposing factor*) AND systematic review or meta-analysis	Include: English, human, Systematic reviews, journal articles, periodicals, 2009-2019<br>Exclude: MEDline records	**24**
Cochrane Reviews	All: Abstract or Title or Keyword<br>(dementia OR alzheimer* OR cognitive impairment* OR cognitive decline OR cognitive function* OR cognitive reserve OR cognitive capacit*) AND (risk factor* OR predictive factor* OR prognostic factor* OR course OR cause OR contributing factor* OR predisposing factor*) AND systematic review or meta-analysis	Include: 2009-2019	**110**
Epistemonikos	Manually: TI/AB:<br>("dementia" OR "alzheimer*" OR "cognitive impairment*" OR "cognitive decline" OR "cognitive function*" OR "cognitive reserve" OR "cognitive capacit*") AND ("risk factor*" OR "predictive factor*" OR "prognostic factor*" OR "course" OR "cause" OR "contributing factor*" OR "predisposing factor*") AND ("systematic review" OR "meta-analysis")	Include:<br>2009-2019, Systematic review<br><br>Exclude:<br>No PMC	**304**

Lenart-Bugla et al., 2022: What do we know about social and non-social factors influencing the pathway from cognitive health to dementia? A systematic review of reviews.

Medline via Ovid	1. dementia.ti,ab,kw. 2. dementia/ 3. alzheimer*.ti,ab,kw. 4. alzheimer disease/ 5. cognitive impairment.ti,ab,kw. 6. cognitive decline.ti,ab,kw. 7. cognitive function*.ti,ab,kw. 8. cognitive capacit*.ti,ab,kw. 9. cognitive reserve.ti,ab,kw. 10. Risk Factors/ 11. risk factor*.ti,ab,kw. 12. predictive factor*.ti,ab,kw. 13. course.ti,ab,kw. 14. cause.ti,ab,kw. 15. prognostic factor*.ti,ab,kw. 16. contributing factor*.ti,ab,kw. 17. predisposing factor*.ti,ab,kw. 18. 1 or 2 or 3 or 4 or 5 or 6 or 7 or 8 or 9 19. 10 or 11 or 12 or 13 or 14 or 15 or 16 or 17 20. 18 and 19 21. 20 not ("economic model*" or "regression model*" or "markov model*" or "statistical model*" or "mathematical model*" or "drug*" or "pharmac*").ti,ab,kw. 22. limit 21 to (english language and systematic review and yr=2009-2019) 23. (animals not (humans and animals)).sh. 24. 22 not 23	limit english language and humans and "systematic review"	**468**
PsycINFO	1. cognitive impairment*.ti,ab,kw.<br>2. dementia/<br>3. Alzheimer's Disease/<br>4. dementia.ti,ab,kw.<br>5. alzheimer*.ti,ab,kw.<br>6. cognitive function*.ti,ab,kw.<br>7. cognitive decline.ti,ab,kw.<br>8. cognitive reserve.ti,ab,kw.<br>9. cognitive capacit*.ti,ab,kw.<br>10. Risk Factors/<br>11. risk factor*.ti,ab,kw.<br>12. predictive factor*.ti,ab,kw.<br>13. course.ti,ab,kw.<br>14. cause.ti,ab,kw.<br>15. prognostic factor*.ti,ab,kw.<br>16. contributing factor*.ti,ab,kw.<br>17. predisposing factor*.ti,ab,kw.<br>18. 1 or 2 or 3 or 4 or 5 or 6 or 7 or 8 or 9<br>19. 10 or 11 or 12 or 13 or 14 or 15 or 16 or 17<br>20. 18 and 19<br>21. limit 20 to ("systematic review" and "english language" and yr="2009 - 2019")<br>22. (animals not (humans and animals)).sh.<br>23. 21 not 22	limit english language and humans and "systematic review", yr= 2009-2019	**282**

Lenart-Bugla et al., 2022: What do we know about social and non-social factors influencing the pathway from cognitive health to dementia? A systematic review of reviews.

An additional search for social factors only

Database	Command	Criteria	Results
CINAHL Complete	All: Abstract<br>Dementia* OR Alzheimer* OR (Cognitive Impairment) OR (Cognitive decline*) OR (Cognitive function*) OR (Cognitive reserve) OR (Cognitive capacit*) AND (risk factor* OR predictive factor* OR prognostic factor* OR course OR cause OR contributing factor* OR predisposing factor*) AND (social) AND systematic review or meta-analysis	Include: English, human, Systematic reviews, journal articles, periodicals, 06.2019-12.2021<br>Exclude: MEDline records	**5**
Cochrane Reviews	All: Abstract or Title or Keyword<br>(dementia OR alzheimer* OR cognitive impairment* OR cognitive decline OR cognitive function* OR cognitive reserve OR cognitive capacit*) AND (risk factor* OR predictive factor* OR prognostic factor* OR course OR cause OR contributing factor* OR predisposing factor*) AND (social) AND systematic review or meta-analysis	Include: 06.2019-12.2021	**10**
Epistemonikos	Manually: TI/AB:<br>("dementia" OR "alzheimer*" OR "cognitive impairment*" OR "cognitive decline" OR "cognitive function*" OR "cognitive reserve" OR "cognitive capacit*") AND (“risk factor*" OR "predictive factor*" OR "prognostic factor*" OR "course" OR "cause" OR "contributing factor*" OR "predisposing factor*") AND (“social”) AND (“systematic review” OR “meta-analysis”)	Include:<br>2019-2021, Systematic review<br><br>Exclude:<br>No PMC	**6**

Lenart-Bugla et al., 2022: What do we know about social and non-social factors influencing the pathway from cognitive health to dementia? A systematic review of reviews.

Medline via Ovid	1. ((dementia or alzheimer* or cognitive impairment* or cognitive decline or cognitive function* or cognitive reserve or cognitive capacit*) and (risk factor* or predictive factor* or prognostic factor* or course or cause or contributing factor* or predisposing factor*) and social and (systematic review or meta-analysis)).ab.<br>2. limit 1 to (english language and "review articles" and humans and yr="2019 - 2021")	limit english language and humans and "systematic review"	**3**
PsycINFO	(**Abstract**: dementia *OR* **Abstract**: alzheimer* *OR* **Abstract**: cognitive impairment* *OR* **Abstract**: cognitive decline *OR* **Abstract**: cognitive function* *OR***Abstract**: cognitive reserve *OR* **Abstract**: cognitive capacit*) *AND* (**Abstract**: risk factor* *OR* **Abstract**: predictive factor* *OR* **Abstract**: prognostic factor* *OR* **Abstract**: course *OR* **Abstract**: cause *OR* **Abstract**: contributing factor* *OR* **Abstract**: predisposing factor*) *AND* **Abstract**: social *AND* (**Abstract**: systematic review *OR***Abstract**: meta-analysis) *AND* **Document Type**: Journal Article *AND* **Methodology**: Systematic Review *OR* Meta Analysis *AND* **Year**: 2019 *To* 2021		**12**

Lenart-Bugla et al., 2022: What do we know about social and non-social factors influencing the pathway from cognitive health to dementia? A systematic review of reviews.

PubMed	((((dementia[Title/Abstract] OR alzheimer*[Title/Abstract] OR cognitive impairment*[Title/Abstract] OR cognitive decline[Title/Abstract] OR cognitive function*[Title/Abstract] OR cognitive reserve[Title/Abstract] OR cognitive capacit*)[Title/Abstract]) AND ((risk factor*[Title/Abstract] OR predictive factor*[Title/Abstract] OR prognostic factor*[Title/Abstract] OR course[Title/Abstract] OR cause[Title/Abstract] OR contributing factor*[Title/Abstract] OR predisposing factor*)[Title/Abstract])) AND (social[Title/Abstract])) AND ((systematic review[Title/Abstract] OR meta-analysis)[Title/Abstract])	Limit to: meta-analysis, systematic review; 06.2019-12.2021	**30**
