# Supplementary material for: What Do We Know about Social and Non-Social Factors Influencing the Pathway from Cognitive Health to Dementia? A Systematic Review of Reviews
Source: Brain Sci. 2022 Sep 8;12(9):1214. doi: 10.3390/brainsci12091214 (PMC9497077; doi:10.3390/brainsci12091214)
Supplement: Supplementary file 1 [file brainsci-12-01214-s001.zip › Table S3. Exclusion criteria.pdf]

Lenart-Bugla et al., 2022: What do we know about social and non-social factors influencing the pathway from cognitive health to dementia? A systematic review of reviews.

Supplementary material
Table S3: Exclusion criteria.

**Other disease**	**Reverse causality**	**Non-human study**	**Pharmacological study**	**Caregiver research**	**Publication type**	**Weak link to research aims**	**No influencing factors**
If the study focuses on different disease(s) and there is no examination whether this disease is associated with i.e. cognitive function/ability, onset/progression of dementia, Alzheimer's and so on.	If the study focuses on the effect/impact of dementia, cognitive decline, Alzheimer's etc. on some kind of variable/factor (i.e. language skills, physical activity, weight loss, apathy). These studies examining issues where dementia/Alzheimer/cognitive decline is causing changes in XY.	If the studies included in the review are solely animal studies (i.e. rats, mice models). If there is a mixed sample with human and animal studies, we will include ("Maybe") this article.	If the study focuses on drug trials or pharmacological interventions for dementia, Alzheimer's, cognitive decline and so on.	If the review examines the situation of/interventions for family and paid carers only (not including people with dementia).	If the study is not a review based on a methodological approach, e.g. a book review, commentary, protocol, non-published dissertation, conference proceeding, conference abstract.	If dementia, Alzheimer's, cognitive decline etc. is mentioned somewhere in the abstract, but the connection to the research aims – factors associated with cognitive decline, dementia, Alzheimer's and so on – is scarcely given.	If the review is focusing on dementia, Alzheimer's, cognitive decline and so on, but is not investigating influencing factors (risk, protective) for i.e. cognitive decline/reserve, onset/progression of dementia/Alzheimer's.
